# Group Cognitive Behavioral Therapy With Virtual Reality Exposure Versus In-Vivo Exposure for Social Anxiety Disorder and Agoraphobia: Underpowered Results From the SoREAL Pragmatic Randomized Clinical Trial

**DOI:** 10.2196/73815

**Published:** 2025-11-03

**Authors:** Benjamin Arnfred, Fatime Zeka, Carsten Hjorthøj, Clas Winding Christensen, Kirsten Stengaard Moeller, Mette Øllgaard Pedersen, Nicole Rosenberg, Lars Clemmensen, Louise Birkedal Glenthøj, Merete Nordentoft

**Affiliations:** 1 Copenhagen Research Center for Mental Health - CORE, Mental Health Center Copenhagen, Copenhagen University Hospital, Gentofte Hospitalsvej 15, Copenhagen, 2900, Denmark, 45 31724603; 2 Department of Psychology, University of Copenhagen, Copenhagen, Denmark; 3 Department of Public Health, University of Copenhagen, Copenhagen, Denmark; 4 Mental Health Centre Copenhagen, Capital Region Psychiatry, Copenhagen, Denmark; 5 Mental Health Centre Stolpegaard, Capital Region Psychiatry, Copenhagen, Denmark; 6 Department for Clinical Medicine, Faculty of Health and Medical Science, University of Copenhagen, Copenhagen, Denmark

**Keywords:** agoraphobia, cognitive behavioral therapy, group therapy, social anxiety disorder, virtual reality

## Abstract

**Background:**

Social anxiety disorder (SAD) and agoraphobia are common, impairing conditions often treated with cognitive behavioral therapy (CBT) conducted in groups. In CBT, exposure therapy is a core element. However, in-vivo exposure therapy is logistically challenging and aversive for both patient and therapist, especially in a group context, often leading to exposure being skipped altogether in clinical practice. Virtual reality exposure (VRE), in which phobic stimuli are presented through immersive virtual reality technology, has shown promise as a flexible alternative to in-vivo exposure. We thus hypothesized that using VRE would result in more overall exposure and more individualized exposure, resulting in statistically significant symptom reduction compared with a group using in-vivo exposure.

**Objective:**

This trial evaluated the efficacy of group CBT with VRE (VR-CBT) versus CBT with in-vivo exposure for treating SAD and agoraphobia in clinical settings.

**Methods:**

In this randomized, parallel-group, assessor-blinded trial, 177 participants with SAD (n=150) or agoraphobia (n=27) as a primary diagnosis were assigned to either VR-CBT (n=81) or traditional CBT (n=96) across 5 Danish mental health outpatient clinics. Both groups received 14 weekly group sessions. The difference between the 2 treatments was that the VR-CBT group received exposure therapy via head-mounted displays (HMDs) displaying 360° videos of anxiogenic situations for individuals with SAD (eg, presenting at work) and agoraphobia (eg, faulty elevator), while the CBT group conducted traditional in-vivo exposure exercises (eg, presenting to the group, using the clinic elevator). Primary outcomes were phobic anxiety reductions, measured by the Liebowitz Social Anxiety Scale and the Mobility Inventory for Agoraphobia at baseline, posttreatment, and 1-year follow-up (from baseline). Secondary outcomes included work and social functioning, depressive symptoms, and quality of life.

**Results:**

Both groups showed significant reductions in primary, secondary, and exploratory outcomes, with no significant differences between groups at posttreatment (*d*=−0.026) and 1-year follow-up (*d*=0.097). Baseline characteristics and attrition rates were balanced across the groups.

**Conclusions:**

Due to insufficient recruitment and substantial missing data, no definitive conclusions can be drawn regarding group differences between VR-CBT and traditional CBT in group settings. The feasibility issues encountered suggest that careful consideration of the benefits and limitations of VR technology is essential before implementation in clinical practice.

## Introduction

Social anxiety disorder (SAD) and agoraphobia are common and impairing mental health conditions. Agoraphobia without comorbid panic disorder has a prevalence of about 1.2%, with most cases (70%) also meeting criteria for panic disorder [1], while the lifetime prevalence of SAD is approximately 4% across low-, middle-, and high-income countries, making it one of the most prevalent mental disorders [2]. Both disorders are associated with decreased quality of life [3], increased suicidality [4], and increased psychiatric comorbidity and typically develop in early adolescence [5], more often in women [1,2], and are often chronic if untreated [6,7]. In Denmark, anxiety disorders rank among the 15 most costly health conditions, due to their early onset, chronicity, and impact on quality of life and productivity [8]. These factors underscore the urgent need for accessible and empirically supported treatments.

Cognitive behavioral therapy (CBT), a research-informed, time-limited psychotherapeutic intervention focused on modifying biased cognitions and dysfunctional behaviors [9,10], is the recommended first-line treatment for SAD and panic disorder with agoraphobia (PD/A) [11-14], demonstrating lasting efficacy [15-21], minimal side effects [15], and being generally preferred over psychopharmacological treatment [22]. Exposure therapy, a key component of CBT for SAD and PD/A, aims to modify patients’ expectations about the likelihood and consequences of a feared outcome by exposing them to feared stimuli [23,24]. However, feared stimuli can sometimes be inaccessible, uncontrollable, or aversive, leading both patients and therapists to avoid exposure therapy [25-28].

Virtual reality (VR) technology offers a controlled and flexible way to expose patients to feared stimuli, potentially overcoming some challenges of traditional exposure therapy [29,30]; for example, as a low-intensity starting option or to expose patients to situations that are not readily available for in-vivo exposure, such as job interviews and car malfunctions. Meta-analyses suggest that virtual reality exposure (VRE)-based treatments are as effective as active control conditions for anxiety disorders [31-33]. However, VRE has primarily been studied in individual therapy rather than group settings and has not been extensively evaluated in naturalistic clinical settings.

In clinical settings, CBT for anxiety disorders is often conducted in groups, as group-based CBT has shown efficacy comparable to individually delivered CBT therapy for both SAD [15,34] and panic disorder with or without agoraphobia [35], while being considered more cost-effective. Based on this, in Danish outpatient psychiatry, SAD and agoraphobia (without comorbid panic disorder) are routinely treated together in “mixed anxiety” CBT groups. The SoREAL trial aimed to evaluate the efficacy of VRE-based CBT for SAD and agoraphobia within this naturalistic, group-based clinical setting. As such, this study was designed as a pragmatic randomized controlled trial, wherein external validity was prioritized by testing interventions under routine clinical conditions with typical patients and standard care procedures. By embedding the intervention within the existing structure of the Danish mental health services and by comparing VR-CBT to a traditional CBT treatment-as-usual intervention, the SoREAL trial aimed to generate evidence not only on efficacy but also on clinical effectiveness (ie, making results as generalizable to real-world practice as possible).

Both treatment arms in the trial included 14 two-hour sessions, of which 7 sessions had scheduled 45 minutes of either VRE or in-vivo exposure. In the VR-CBT arm, patients were handed personal head-mounted displays and could individually choose to be exposed to a range of immersive virtual scenarios relevant to their disorder, including social interactions (eg, public speaking, small talk, meetings) and agoraphobic situations (eg, crowded bus, supermarket, elevator). In the CBT arm, patients were asked to conduct a range of exposure exercises including small-talk roleplay, taking the clinic elevator and pressing stop, inducing nausea (eg, through spinning on the spot), and presenting in the group, which all required preparation and often requires the whole group to conduct the same exercise. We therefore expected the VRE format to allow for more tailored and higher dosages of exposure than in-vivo exposure therapy, and thus hypothesized that VR-CBT would result in greater reductions in phobic anxiety compared to traditional CBT.

## Methods

### Design

We conducted a 1:1 randomized, assessor-blinded, parallel-group superiority trial across 5 outpatient psychotherapeutic clinics offering standardized anxiety group therapy within Danish mental health services. Consolidated Standards of Reporting Trials guidelines were followed in reporting this trial [36].

We estimated that 302 participants were needed to detect the minimal clinically relevant differences in phobic anxiety severity reductions between groups [37]. Participant recruitment began in February 2019 and ended prematurely in December 2023 due to recruitment and feasibility issues, resulting in a sample size of 177 (81 in the VR-CBT arm and 96 in the CBT arm). These issues are elaborated in the discussion section.

### Design Changes After Trial Registration

The SoREAL trial was initially designed to investigate the efficacy of group VR-CBT for SAD (ICD code F40.1), and this design was registered at ClinicalTrials.gov as NCT03845101 before inclusion began. However, to address severe recruitment problems, we implemented several changes to the trial’s design. We revised the inclusion criteria: removing a Liebowitz Social Anxiety Scale (LSAS) [38] score requirement of >65 and adding agoraphobia (ICD code F40.0) as a possible primary diagnosis. Therapy manuals and VR software were modified with agoraphobia-specific modules and VR environments. The primary outcome measure was changed to percentage of maximum points (POMP)-transformed total scores of the LSAS for patients with SAD and the Mobility Inventory for Agoraphobia (MIA) [39] for patients with agoraphobia. POMP transforming 2 different measures of anxiety allowed us to include patients with different primary diagnoses in the same analysis [40,41]. Finally, we expanded from 2 to 5 clinical sites and adjusted randomization to allocate therapy groups (rather than individual participants), stratified by site, to minimize wait times and account for the geographical distance between sites. The randomization module was updated in March 2020 after 40 participants had been individually randomized. The final trial design is also described in the published trial protocol [37]. The design changes did not entail any changes to the statistical analysis plan (beyond the changed primary outcome). However, we did add exploratory diagnose-specific subgroup analysis of both MIA and LSAS. These showed that both subgroups had similar effect sizes between treatment arms, suggesting that the inclusion of agoraphobia did not meaningfully alter the overall treatment effect.

### Participants

Patients were recruited from 5 outpatient clinic sites while on the waitlist for treatment. Eligible participants had a primary diagnosis of SAD (ICD code F40.1) or agoraphobia (ICD code F40.0), were aged 18‐75 years, had adequate proficiency in Danish, and were willing and able to provide informed consent. Patients were excluded if they had drug or alcohol dependence. If a patient met diagnostic criteria for both SAD and agoraphobia, 1 diagnosis was designated as primary based on the referral from the regional Center for Visitation and Diagnostics, which assesses and refers patients to outpatient psychiatric care in Denmark. Assessors at the Center for Visitation and Diagnostics are clinical psychologists and medical doctors trained in broad psychiatric assessment and supervised regularly by clinical specialists (eg, psychiatrists). At the baseline research interview, the primary diagnosis was confirmed using the Mini-International Neuropsychiatric Interview version 7 [42], conducted by researchers (psychology graduate students or psychologists) who received training in the use of the Mini-International Neuropsychiatric Interview and participated in structured hierarchical interrater sessions every 3 weeks, led by the first author.

### Randomization and Blinding

Therapy groups were randomly assigned (1:1) to either VR-CBT mixed anxiety groups or CBT with in-vivo exposure mixed anxiety groups. Randomization was performed using REDCap [43] by a trial coordinator uninvolved in data collection, who informed the relevant clinicians via email or telephone. Allocation tables were generated by a statistician using an online tool [44] with varying block sizes of 2 and 4 and stratification by treatment site. Trial assessors were blinded to group allocation, and if unblinded, another assessor conducted the assessment.

### Procedures

Patients were assessed before treatment, approximately 2 weeks after treatment, and 12 months after beginning treatment by trained assessors. The 12-month follow-up was included to evaluate the durability of therapeutic gains [21]. Assessments took place at the clinics where patients received treatment or via telephone if necessary due to COVID-19 restrictions. Eligibility was confirmed by assessors during the baseline interview. Self-reported questionnaires were answered by hand (on printed copies) or online using a secure link. Both interventions followed a standardized structure and included the same core elements: 1 hour of individual therapy in preparation for group sessions; 14 weekly 2-hour group therapy sessions; and optional components consisting of next-of-kin involvement, pharmacological treatment planning, physical therapy, and coordination with social services. The group therapy in both interventions was manualized and based on established CBT approaches to treating anxiety [45-48]. The manual was flexible, allowing clinicians to change the order of sessions. However, the amount of time allocated for exposure in both interventions was fixed, encompassing all related processes such as setup, transport, and preparation. Therapy groups consisted of 8-9 patients and were led by 2 clinicians trained in CBT (eg, psychologists, medical doctors, or physiotherapists trained in psychotherapy). Acute supplementary individual sessions were also available in both interventions. In 7 sessions of the 14 sessions, 45 minutes were allocated to either VR or in-vivo exposure exercises. Both interventions included homework consisting of in-vivo exposure tasks between sessions and 1 session of “out-of-clinic” in-vivo exposure (eg, taking the bus or going shopping). The exposure exercises aimed to foster adaptive responses to anxiety-provoking situations, reinforce cognitive restructuring, train general cognitive strategies (eg, identifying negative automatic thoughts), and build social skills. The cognitive therapy strategies used in nonexposure sessions included psychoeducation, cognitive restructuring, shifting self-focused attention, and relapse prevention. The interventions differed only in the delivery method of exposure therapy.

### VR Exposure

Each patient received an Oculus Go head-mounted display and high-quality sound-blocking headphones for use throughout treatment. The VR environments were high-resolution, 360° stereoscopic films depicting salient, fear-inducing situations for SAD and agoraphobia. Patients chose from 13 scenarios with 4-6 scenes of increasing difficulty. Scenarios were designed specifically for either SAD (eg, party, small talk during lunch, presenting at work) or agoraphobia (eg, crossing a bridge while carpooling with strangers, taking a commercial airplane, leaving your apartment). However, many scenes had elements that could trigger both fears (eg, attending a lecture at a university). Scenes transitioned to a looped, action-free sequence in the same environment to allow habituation as needed. Patients were introduced to the VR equipment and guided to select scenarios relevant to their anxieties. Exposure exercises were completed individually in the therapy room, with sessions typically including 1 or 2 breaks for group discussions. The exposure platform was developed collaboratively over 16 months with CBT experts, lived experience experts, and VR developers. Lived experience experts provided input on anxiety levels and scenario relevance, which guided adjustments. Details about the VR environments are available in Multimedia Appendix 1.

### In-Vivo Exposure

In-vivo exposure exercises included small talk (among patients), behavioral experiments within the clinic (eg, asking the receptionist for directions), role play (eg, job interviews), inducing nausea through spinning on the spot (eg, exposure to fear of loss of control through vomiting), taking a small clinic elevator (eg, exposure to fear of being unable to escape a situation), increasing heart rate through physical activity (eg, exposure to fear of passing out or having a heart attack), sitting far away from exits in the group therapy room (eg, exposure to fear of being unable to escape a situation), drinking water and coffee and holding off going to the bathroom (eg, exposure to fear of loss of control through incontinence), and public speaking exercises (eg, talking about a hobby in the group). Detailed intervention descriptions are available in the trial protocol [37].

### Outcomes

Primary diagnoses and comorbidities were assessed with the Mini-International Neuropsychiatric Interview version 7 [42]. The primary outcome was a reduction in phobic anxiety, measured with POMP-transformed total scores of the LSAS [38] (clinician-rated) for patients with SAD and the MIA [39] for patients with agoraphobia.

Secondary outcomes included fear of negative evaluation, measured with the Brief Version of the Fear of Negative Evaluation Scale [49]; depression symptoms, measured with the 6-item version of the Hamilton Depression Rating Scale [50]; work and social functioning, measured with the Work and Social Adjustment Scale [51]; treatment satisfaction, measured with the Client Satisfaction Questionnaire [52]; and quality of life, measured with the WHO-5 Well-Being Index [53]. SAD treatment response was defined as an LSAS score below 50 or a reduction of 15 points, and remission was defined as an LSAS score below 25. For agoraphobia, treatment response was defined as an MIA score below 2 or a reduction of 0.5 points, and remission was defined as an MIA score below 1.5, assessed posttreatment and at follow-up.

Exploratory outcomes included social functioning, measured with the Personal and Social Performance Scale [54]; substance and alcohol use over the last month, measured with Timeline Followback [55]; self-efficacy, measured with the General Self-Efficacy Scale [56]; working alliance, measured with the Working Alliance Inventory [57]; SAD symptom severity for patients with SAD as a primary diagnosis, measured with the LSAS (clinician-rated); and agoraphobia symptoms for patients with agoraphobia as a primary diagnosis, measured with the MIA.

Other measures included simulation sickness from VR, assessed using the Simulation Sickness Questionnaire [58] after each exposure session. Deterioration and adverse effects of psychotherapy on SAD symptoms were defined as an increase of 6.8 points or more on the LSAS total score, while for agoraphobia, deterioration was defined as an increase of 0.3 points or more on the MIA total score, assessed posttreatment and at follow-up. These thresholds reflect the smallest clinically meaningful change, corresponding to a Cohen *d* of 0.33 based on prior studies' standard deviations [59,60]. Treatment fidelity was evaluated using a therapist self-report questionnaire administered 5 times throughout therapy, consisting of 6 Likert-style questions (ranging from 1 to 5) and questions about time spent on exposure therapy.

### Statistical Analysis

We aimed to enroll 302 patients based on the following parameters: an alpha of .05; 80% power; and a standard deviation of 21 to detect a difference of 6.8 on the LSAS, corresponding to a Cohen *d* of 0.33. After expanding inclusion to patients with agoraphobia, we retained the target N of 302, even though the lower variability of the MIA would have reduced the required sample size. All analyses were conducted using an intention-to-treat approach, with tests performed at a 2-tailed 5% level of significance. Missing data were handled using multiple imputations (m=100), with predictors in the imputation model including baseline and posttreatment scores of primary, secondary, and exploratory outcomes; primary diagnosis (SAD or agoraphobia); degree of comorbidity; and session attendance. Group differences were examined using analysis of covariance (ANCOVA), with primary diagnosis, degree of comorbidity, and baseline severity as covariates. For POMP-transformed outcomes and analyses of specific diagnostic groups, primary diagnosis was not included as a covariate. Assumptions for ANCOVA were checked as follows: normality of the dependent variable via visual inspection of Q-Q plots, homogeneity of variance using Levene test, and homogeneity of regression slopes with univariate analysis of variance. Baseline severity was excluded as a covariate for posttreatment analyses of the Fear of Negative Evaluation and Work and Social Adjustment Scales, and posttreatment severity was excluded as a covariate for follow-up analysis of the Hamilton Depression Rating Scale due to nonhomogeneity of regression slopes. For dichotomous outcomes, multiple logistic regression analyses were performed using primary diagnosis, comorbidity, and baseline severity as predictors. Model fit was assessed using the Hosmer-Lemeshow test, and multicollinearity was evaluated using tolerance values and variance inflation factors. Due to the small number of drug use incidents (<5 in both groups), drug use was not analyzed. To examine the robustness of the results, we conducted additional post hoc analyses using multilevel models, ANCOVAs on change scores, and random forest imputation. These analyses supported the main findings and confirmed the overall pattern of results. See Multimedia Appendix 2.

### Ethical Considerations

The SoREAL trial received approval from the Regional Ethics Committee of Zealand (H-6-2013-015) and the Danish Data Protection Agency (RHP2014-009-02670). All participants were informed about the study procedures and provided written informed consent prior to participation. Participants were not compensated. The individuals appearing in the supplemental material are professional actors who were hired for this study and were compensated 584.5 USD. Written consent has been obtained from all actors for the use of their images in this publication.

## Results

### Patient Characteristics

**Figure 1.**
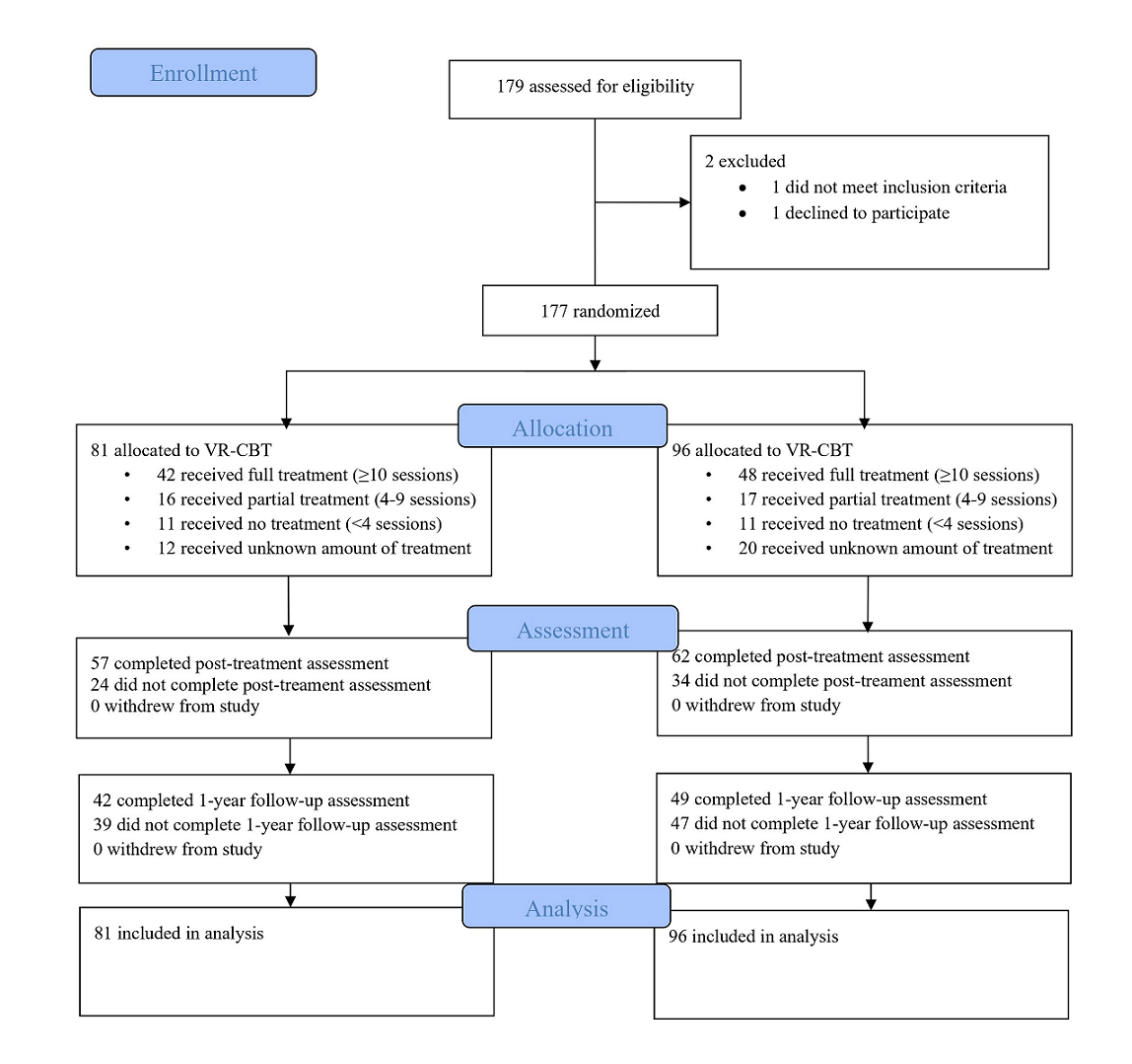
Recruitment and assessment flow of the trial. VR-CBT, virtual reality cognitive behavioral therapy.

A total of 177 participants were analyzed, with 81 randomized to VR-CBT and 96 to CBT. Figure 1 illustrates the recruitment and assessment flow of the trial. Sociodemographic and clinical characteristics were balanced between the groups (Table 1).

**Table 1.** Baseline characteristics.

Characteristic	VR-CBT (n=81)	CBT (n=96)
Age (years), mean (SD)	29.5 (9.7)	28.5 (9.6)
Sex, n (%)		
Male	34 (42)	44 (45.8)
Female	46 (56.8)	51 (53.1)
Other	1 (1.2)	1 (1)
Education, n (%)		
Primary education	21 (28.4)	17 (20.5)
Secondary/vocational education	28 (37.8)	38 (45.8)
Postsecondary education	23 (31.1)	28 (33.7)
Other	2 (2.7)	0 (0)
Civil status, n (%)		
Partnered	27 (36)	27 (31)
Not partnered	45 (60)	53 (60.9)
Other	3 (4)	7 (8)
Previous psychiatric treatment, n (%)		
No	12 (16)	26 (29.5)
Yes	63 (84)	62 (70.5)
Number of psychiatric comorbidities, n (%)		
0	32 (39.5)	35 (36.5)
1	21 (25.9)	32 (33.3)
2	18 (22.2)	17 (17.7)
3	10 (12.3)	12 (12.5)
Currently in psychopharmacological treatment, n (%)		
No	32 (39.5)	41 (42.7)
Yes	49 (60.5)	55 (57.3)
Primary diagnosis, n (%)		
Social anxiety disorder	71 (87.7)	79 (82.3)
Agoraphobia	10 (12.3)	17 (17.7)

Missing data rates for POMP scores were substantial at posttreatment (VR-CBT: 29.6%, CBT: 35.4%) and follow-up (VR-CBT: 48.1%, CBT: 49.0%). The only identifiable reason for missing data was participants not responding to contact attempts or failing to attend scheduled assessment sessions, with no specific reasons reported by the participants. Missing data rates were not significantly different between groups but were significantly associated with older age (*P*=.03), having a primary diagnosis of agoraphobia, and fewer sessions attended (*P*<.001) (Table 2). On average, participants attended 9 therapy sessions in the VR-CBT group and 9.3 therapy sessions in the CBT group. A total of 22 (12.4%) of participants received no treatment (<4 sessions), 33 (18.6%) received partial treatment (4‐9 sessions), and 110 (62.1%) completed treatment (10+ sessions). Engagement was comparable across the groups: 48 (50%) in the CBT group and 42 (51.8%) in the VR-CBT group completed treatment.

**Table 2.** Attrition analysis^a^.

Variables	Posttreatment attrition (primary outcome)		Follow-up attrition (primary outcome)	
	*P* value	Effect	*P* value	Effect
Group assignment	.41	—^b^	.91	—
Primary diagnosis	.02	Agoraphobia as primary diagnosis associated with attrition	.43	—
Number of psychiatric comorbidities	.23	—	>.99	—
Previous psychiatric treatment	.25	—	.30	—
Sex	.76	—	.13	—
Age	.03	Older age associated with attrition	.10	—
Baseline anxiety severity	.46	—	.30	—
Number of sessions attended	<.001	Attending fewer sessions associated with attrition	<.001	Attending fewer sessions associated with attrition

a Continuous variables were analyzed using independent *t* tests (2-sided). Categorical variables were assessed using Pearson’s *Χ*2 tests (asymptotic significance).

b —: No statistically significant effect.

### Primary Outcomes

Both groups showed reductions in phobic anxiety severity. From baseline to posttreatment, the VR-CBT group improved by an estimated 11.3 points and the CBT group by 12.0 points (Table 3). The between-group difference in change scores was not statistically significant (*B*=–1.33, 95% CI –6.38 to 3.73, *P*=.61, Cohen *d*=–0.03) (Table 4). Adjusted posttreatment means were 47.4 (SE=2.3) for VR-CBT and 46.9 (SE=2.4) for CBT (Table 3). From posttreatment to follow-up, the VR-CBT group showed an additional improvement of 3.8 points, whereas the CBT group improved by 1.7 points (Table 3). The between-group difference in change scores was not statistically significant (*B*=1.62, 95% CI –2.74 to 5.98, *P*=.47, Cohen *d*=0.10) (Table 4).

**Table 3.** Adjusted means of outcome measures^a^.

	VR-CBT			CBT		
Outcome measure	Baseline	Posttreatment	Follow-up	Baseline	Posttreatment	Follow-up
Primary outcomes						
Phobic anxiety severity (LSAS^b^ and MIA^c^ [POMP^d^ transformed]), adjusted mean (SE; 95% CI)	58.78 (1.68; 55.50-62.07)	47.45 (2.30; 42.88-52.02)	43.63 (1.82; 40.05-47.21)	58.83 (1.53; 55.83-61.82)	46.88 (2.37; 42.18-51.59)	45.25 (1.75; 41.80-48.69)
Secondary outcomes						
Fear of negative evaluation (FNES^e^), adjusted mean (SE; 95% CI)	49.65 (0.82; 48.05-51.25)	44.69 (1.27; 42.20-47.17)	44.47 (1.46; 41.59-47.35)	49.15 (0.75; 47.68-50.62)	44.01 (1.29; 41.49-46.54)	44.96 (1.33; 42.35-47.58)
Depressive symptoms (HAM-6^f^), adjusted mean (SE; 95% CI)	6.65 (0.37; 5.92-7.39)	4.84 (0.48; 3.91-5.77)	5.14 (0.62; 3.93-6.35)	6.38 (0.34; 5.70-7.05)	5.12 (0.46; 4.22-6.02)	5.35 (0.59; 4.20-6.50)
Work and social adjustment (WSAS^g^), adjusted mean (SE; 95% CI)	4.32 (0.17; 3.98-4.66)	3.77 (0.25; 3.28-4.26)	3.48 (0.30; 2.88-4.07)	4.25 (0.16; 3.94-4.56)	3.55 (0.23; 3.09-4.01)	3.46 (0.28; 2.90-4.01)
Quality of life (WHO-5^h^), adjusted mean (SE; 95% CI)	35.56 (1.89; 31.86-39.26)	41.56 (2.99; 35.68-47.44)	42.86 (3.59; 35.78-49.94)	34.17 (1.76; 30.73-37.62)	43.44 (2.80; 37.95-48.94)	43.10 (2.98; 37.24-48.97)
Acceptability and satisfaction with treatment (CSQ^i^), adjusted mean (SE; 95% CI)	N/A^j^	24.77 (0.68; 23.44-26.10)	N/A	N/A	24.84 (0.71; 23.44-26.23)	N/A
Exploratory outcomes						
Social functioning (PSP^k^), adjusted mean (SE; 95% CI)	63.54 (1.25; 61.10-65.99)	67.67 (1.34; 65.05-70.29)	69.55 (1.78; 66.06-73.04)	63.43 (1.14; 61.19-65.67)	68.93 (1.28; 66.42-71.43)	69.29 (1.62; 66.11-72.47)
Alcohol use (TLFB^l^ (units of 12 g of alcohol last 30 d)), adjusted mean (SE; 95% CI)	10.48 (2.08; 6.42-14.55)	5.01 (3.51-7.03)	5.65 (3.81-8.22)	11.14 (1.89; 7.44-14.85)	6.32 (4.56-8.62)	6.68 (4.74-9.28)
Self-belief of coping (GSE^m^), adjusted mean (SE; 95% CI)	21.86 (0.61; 20.66-23.06)	23.99 (0.77; 22.48-25.50)	24.49 (0.95; 22.62-26.36)	22.06 (0.58; 20.93-23.19)	23.80 (0.71; 22.41-25.19)	24.34 (0.91; 22.55-26.12)
Working alliance (WAI^n^), adjusted mean (SE; 95% CI)	N/A	3.58 (0.12; 3.35-3.81)	N/A	N/A	3.60 (0.12; 3.38-3.83)	N/A
Social anxiety symptoms (participants with primary diagnosis of SAD^o^) (LSAS), adjusted mean (SE; 95% CI)	89.48 (2.33; 84.92-94.04)	68.96 (2.76; 63.55-74.38)	66.47 (2.67; 61.24-71.71)	87.09 (2.21; 82.77-91.42)	70.98 (2.72; 65.65-76.32)	68.18 (2.66; 62.96-73.40)
Agoraphobia symptoms (participants with primary diagnosis of agoraphobia) (MIA), adjusted mean (SE; 95% CI)	2.67 (0.35; 1.98-3.35)	2.18 (0.31; 1.57-2.79)	2.15 (0.32; 1.51-2.79)	3.00 (0.23; 2.54-3.46)	1.99 (0.21; 1.57-2.40)	2.23 (0.24; 1.77-2.70)

a Data are pooled from 100 imputations. Covariates are identical to the ANCOVA models.

b LSAS: Leibowitz Social Anxiety Scale.

c MIA: Mobility Inventory for Agoraphobia.

d PMOP: percentage of maximum points.

e FNES: Fear of Negative Evaluation Scale.

f HAM-6: Hamilton Depression Scale.

g WSAS: Work and Social Adjustment Scale.

h WHO-5: World Health Organization Wellbeing Index.

i CSQ: Client Satisfaction Questionnaire.

j N/A: not applicable.

k PSP: Personal and Social Performance Scale.

l TLFB: Timeline Followback for alcohol consumption.

m GSE: General Self-Efficacy Scale.

n WAI: Working Alliance Inventory.

o SAD: Social Anxiety Disorder.

**Table 4.** Primary, secondary, and exploratory results from analysis of covariance (ANCOVA)^a^.

	Posttreatment				1-year follow-up			
Outcome measure	*B* (SE; 95% CI)	*t*	*P* value	Cohen *d*	*B* (SE; 95% CI)	*t*	*P* value	Cohen *d*
Primary outcomes								
Phobic anxiety severity^b^ (LSAS^c^ and MIA^d^ POMP^e^ transformed)	–1.328 (2.579; –6.384 to 3.728)	–.515	.61	–0.026	1.619 (2.222; –2.740 to 5.977)	0.728	.47	0.097
Secondary outcomes								
Fear of negative evaluation^f^ (FNES^g^)	–0.675 (1.703; –4.015 to 2.666)	–0.396	.69	0.27	0.493 (1.660; –2.766 to 3.753)	0.297	.77	0.03
Depressive symptoms^h^ (HAM-6^i^)	0.279 (0.639; –0.975 to 1.533)	0.437	.66	0.063	0.214 (0.728; –1.214 to 1.643)	0.294	.77	0.037
Work and social adjustment^j^ (WSAS^k^)	–0.214 (0.318; –0.839 to 0.411)	–0.673	.50	–0.094	–0.22 (0.335; –0.679 to 0.635)	–0.065	.95	–0.007
Quality of life (WHO-5^l^)	1.883 (3.755; –5.488 to 9.254)	0.501	.62	0.069	0.245 (3.469; –6.563 to 7.053)	0.071	>.99	0.008
Acceptability and satisfaction with treatment (CSQ^m^)	0.069 (0.869; –1.636 to 1.774)	0.080	.94	0.011	N/A^n^	N/A	N/A	N/A
Exploratory outcomes								
Social functioning (PSP^o^)	1.257 (1.783; –2.241 to 4.754)	0.705	.48	0.102	–0.262 (2.024; –4.233 to 3.709)	–0.129	.90	–0.016
Alcohol use (TLFB^p^ [units of 12 g of alcohol last 30 d])	0.215 (0.197; –0.173 to 0.788)	0.993	.32	0.145^j^	0.154 (0.201; –0.221 to 0.711)	0.714	.48	0.097^j^
Self-belief of coping (GSE^q^)	–0.194 (0.932; –2.024 to 1.635)	–0.209	.84	−0.027	–0.152 (1.008; –2.131 to 1.828)	–0.150	.88	–0.017
Working alliance^f^ (WAI^r^)	0.023 (0.152; –0.276 to 0.321)	0.149	.88	0.018	N/A	N/A	N/A	N/A
Social anxiety symptoms^b^ (participants with primary diagnosis of SAD)^s^ (LSAS)	2.017 (3.829; –5.489 to 9.523)	0.527	.60	0.085	1.708 (3.358; –4.881 to 8.296)	0.509	.61	0.085
Agoraphobia symptoms^b^ (participants with primary diagnosis of agoraphobia) (MIA)	–0.193 (0.332; –0.846 to 0.459)	–0.582	.56	0.114	0.82 (0.381; –0.666 to 0.30)	0.215	.83	0.082

a Treatment effects (ie, the interaction of time and treatment condition) are shown. Positive B values indicate an effect in favor of the CBT group. Results are pooled from 100 imputations. Unless otherwise noted, covariates are primary diagnosis, comorbidity, and baseline severity. For all follow-up analysis, posttreatment severity was added as a covariate. Phobic anxiety severity included sessions attended as a covariate.

b Primary diagnoses not included as a covariate.

c LSAS: Leibowitz Social Anxiety Scale.

d MIA: Mobility Inventory for Agoraphobia.

e POMP: percentage of maximum points.

f Baseline severity violated the assumption of homogeneity of regression slopes, thus not included as a covariate.

g FNES: Fear of Negative Evaluation Scale.

h Posttreatment severity violated the assumption of homogeneity of regression slopes, thus not included as a covariate.

i HAM-6: Hamilton Depression Scale.

j Calculated with logarithmic means and SDs.

k WSAS: Work and Social Adjustment Scale.

l WHO-5: World Health Organization Wellbeing Index.

m CSQ: Client Satisfaction Questionnaire.

n N/A: Not applicable.

o PSP: Personal and Social Performance Scale.

p TLFB: Timeline Followback for Alcohol Consumption.

q GSE: General Self-Efficacy Scale.

r WAI: Working Alliance Inventory.

s SAD: Social Anxiety Disorder.

### Secondary Outcomes

Depressive symptoms decreased in both groups, with no statistically significant between-group differences (posttreatment adjusted means: VR-CBT=4.8, SE=0.4; CBT=5.1, SE=0.4; *P*=.66; follow-up adjusted means: VR-CBT=5.1, SE=0.6; CBT=5.3, SE =0.5; *P*=.77). Fear of negative evaluation also decreased similarly in both groups (posttreatment adjusted means: VR-CBT=44.69, SE=1.27; CBT=44.01, SE=1.29; *P*=.69; follow-up adjusted means: VR-CBT=44.47, SE=1.46; CBT=44.96, SE=1.33; *P*=.77) (Tables3  4). Work and social adjustment scores improved slightly in both groups, with no significant between-group differences (posttreatment adjusted means: VR-CBT=3.7, SE=0.2; CBT=3.5, SE=0.2; *P*=.50; follow-up adjusted means: VR-CBT=3.4, SE=0.3; CBT=3.4, SE=0.2; *P*=.95). Quality of life also improved, but differences between groups were not statistically significant (posttreatment adjusted means: VR-CBT=41.5, SE=2.9; CBT=43.4, SE=2.8; *P*=.62; follow-up adjusted means: VR-CBT=42.8, SE=3.5; CBT=43.1, SE=2.9; *P*=.99) (Tables3  4). At posttreatment, the odds of treatment response did not differ significantly between groups (*P*=.65), and no statistically significant differences were observed for the odds of remission at posttreatment (*P*=.49) or follow-up (*P*=.51) (Table 5).

**Table 5.** Results of logistic regression analysis on categorical outcomes^a^.

	Posttreatment			Follow-up		
Outcome measures	B (SE)	*P* value	Exp *B* (95% CI)	*B* (SE)	*P* value	Exp *B* (95% CI)
Secondary outcomes						
Response to treatment (LSAS^b^ below 50 or a 15 drop/MIA^c^ below 2 or a 0.5 drop)	0.157 (0.346)	.65	1.170 (0.593-2.307)	N/A^d^	N/A	N/A
Remission (LSAS below 25/MIA below 1.5)	–0.449 (0.654)	.49	0.638 (0.177-2.300)	–0.625 (0.953)	.51	0.535 (0.083-3.472)
Other measures						
Deterioration of phobic anxiety symptoms (6.8 or higher increase in LSAS score/0.3 or higher increase in MIA score)	0.224 (0.538)	.68	1.251 (0.435-3.594)	–0.285 (0.436)	.51	0.752 (0.319-1.771)

a Positive *B* values indicate higher odds of the outcome for the CBT group compared to the VR-CBT group. Results are pooled from 100 imputations. Predictors included in the models were baseline severity, comorbidity, and primary diagnoses.

b LSAS: Liebowitz Social Anxiety Scale.

c MIA: Mobility Inventory for Agoraphobia.

d N/A: Not applicable.

### Exploratory Outcomes

Social functioning and self-belief in coping improved in both groups, with no statistically significant between-group differences at posttreatment or follow-up (posttreatment social functioning adjusted means: VR-CBT=67.6, SE=1.3; CBT=68.9, SE=1.2, *P*=.48; follow-up social functioning adjusted means: VR-CBT=69.5, SE=1.7; CBT=69.2, SE=1.6; all *P*=.90). Similarly, no statistically significant between-group differences were found for alcohol use or treatment satisfaction (Tables3  4).

### Other Measures

No statistically significant group differences were observed regarding deterioration of phobic anxiety symptoms (Table 5). The average total exposure time per therapy course was not statistically significantly different between groups (*P*=.09): 85 minutes for VR-CBT groups (SD 76.5) and 120.7 minutes for CBT groups (SD 76.5), which were overall less than a third of the 360 minutes specified in the treatment manuals. Therapist-reported fidelity scores did not differ significantly between groups (*P*=.24), with a mean of 4.0 (SD 0.3) for VR-CBT and 4.1 (SD 0.5) for CBT, suggesting that therapists adhered closely to the manualized treatment protocols. Among participants receiving VR-CBT, the mean simulation sickness score was high, with an average score of 36.2 (SD 27.9) across 51 participants. There were no reported serious adverse events or reactions.

## Discussion

### Principal Findings

This trial evaluated the effects of group-based VR-CBT with 360° video exposure versus traditional in-vivo group CBT for treating SAD and agoraphobia in a naturalistic clinical setting. Both groups showed reductions in symptoms, with no statistically significant differences observed in primary, secondary, or exploratory outcomes. However, due to the limited statistical power of the study, these results must be interpreted with caution. We cannot conclude that VR-CBT and traditional CBT are equally effective, nor can we exclude the possibility of differences in efficacy between the two treatments. Nonetheless, this pragmatic trial offers a unique contribution by evaluating VR-CBT as it might be delivered in routine care, embedded within existing services, by local clinicians, and under everyday constraints. As such, this trial provides valuable insights into the practical implementation of VR-CBT in naturalistic clinical settings and highlights critical factors influencing its feasibility and acceptability.

### Failure to Reach N

Being fully embedded in the Danish Mental Health Services, the SoREAL trial could only recruit patients referred by their general practitioner to the standardized anxiety treatment package. Based on clinical intake and experience from previous trials at the 2 initial sites, we expected to reach N in 36 months. However, changes to general practitioner guidelines for psychiatric referrals, designed to reduce the case load of psychotherapeutic outpatient hospitals, resulted in most patients with SAD being referred to practicing psychologists instead. To offset the slower-than-anticipated recruitment rate, we expanded the study to include 3 additional clinic sites, which also entailed the design changes described elsewhere.

Shortly after including the new sites, Denmark entered lockdown due to the COVID-19 pandemic. This meant that psychotherapeutic treatment was conducted via telephone or individually. A notable drop in referrals to treatment sites also occurred, possibly because SAD and agoraphobia symptoms are less functionally impairing during a national lockdown.

After the lockdown ended, the clinical sites decided not to continue with the trial for several reasons. The primary concern raised by the clinics was the ethicality of continuing a treatment perceived as unsuitable for the population and the group format. These concerns stemmed from observations that patients found VR distressing in a group setting and struggled to focus on VR scenarios due to the social dynamics within the therapy room. Clinicians also raised concerns about the lack of interactivity in the virtual environments, which made it difficult to expose patients to core SAD fears, such as conversation. These concerns are consistent with prior findings [61] and align with the notion that therapeutic context, patient characteristics, and VR characteristics can influence the experience of VR environments [62]. Group settings, in particular, may introduce stressors like performance anxiety, which could potentially diminish immersion and thus the therapeutic value of VR environments. Addressing these concerns may require the use of VR technology that accounts for group settings and the need for flexible social interactions. Examples include multi-user VR in which all patients engage in the same shared virtual environments; augmented reality, where the therapy room remains visible but virtual objects are overlaid to simulate therapeutic scenarios; or large language model–based avatars to engage in conversation.

Beyond this, staff turnover limited clinicians’ ability to gain experience with VR-CBT. Overall, 39 therapists delivered VR-CBT, most of whom could only receive very limited training in conducting VRE due to time constraints. Frequent technical malfunctions and limited time for in-session troubleshooting also impacted feasibility. These factors likely contributed to the low mean exposure time per VR-CBT group (85 min). Still, the fact that dozens of therapists across multiple sites were able to deliver VR-CBT in suboptimal real-world conditions illustrates the intervention’s potential for scalability if appropriately supported. This also reflects a key strength of the study design: by relying on routine clinic staff, the trial offers an ecologically valid picture of what implementation might look like, and how effective the treatment might be, outside controlled research settings. Lastly, the extended inclusion period strained the resources of the clinics, which had agreed to participate for limited periods (eg, 2 y) and had obligations to other clinical trials. The factors that led clinics to opt out of the trial are important to consider for future studies examining VR-based psychotherapy in naturalistic clinical settings. They also underscore the importance of pilot studies for both design and intervention feasibility [63], which could have identified challenges related to recruitment rates, the group format, technical issues, and clinician training requirements before conducting the full-scale randomized controlled trial.

Beyond incomplete recruitment, the trial’s substantial missing data further impede interpretation, as data are likely missing due to factors associated with the intervention (ie, it is not missing at random [64]). As such, the missing data likely impact the accuracy of the results. However, since data are missing equally across both groups and the variables associated with missing data are balanced between groups, any influence on the results is likely comparable for both groups.

### Limitations

This study has some limitations. Insufficient recruitment and missing data prevent conclusions about group differences. VR acceptability, technical issues, and limited training impacted VR-CBT implementation. The generalizability of findings is limited due to the naturalistic study design.

### Conclusions

Due to the study’s underpowered analysis and substantial missing data, we are unable to draw definitive conclusions about group differences between VR-CBT and CBT in group therapy. The feasibility challenges encountered underscore the need to carefully evaluate both benefits and limitations of VR technology before clinical implementation. The study contributes novel knowledge on the clinical integration of VR in routine care and highlights conditions under which such technology may or may not thrive. Future research could explore VR technologies that account for the group therapy context, such as multiuser VR or augmented reality.

## Supplementary material

10.2196/73815 Multimedia Appendix 1 Descriptions and screenshots of virtual environments.

10.2196/73815 Multimedia Appendix 2 Post hoc analyses.

10.2196/73815 Checklist 1 CONSORT-eHEALTH checklist (V 1.6.1).
