# Supplementary material for: Group Cognitive Behavioral Therapy With Virtual Reality Exposure Versus In-Vivo Exposure for Social Anxiety Disorder and Agoraphobia: Underpowered Results From the SoREAL Pragmatic Randomized Clinical Trial
Source: JMIR Ment Health. 2025 Nov 3;12:e73815. doi: 10.2196/73815 (PMC12582524; doi:10.2196/73815)
Supplement: Multimedia Appendix 1 [file mental-v12-e73815-s001.pdf]

Supplemental file 1 – Descriptions and screenshots of virtual environments used in the SoREAL trial.

All identifiable persons depicted are paid actors.

| | |
|---|---|
| **Environment 1 – Supermarket**<br><br>Scene 0. Loop – Standing by the register. The supermarket is empty. Link to YouTube<br><br>Scene 1. 1:00 – Loop of scene 0. Standing in line. A man asks if you would use a ware separator. Link to YouTube<br><br>Scene 2. 1:39 – Loop of scene 0. Intimidating man cuts in line. Person in line is upset. You have forgotten to weigh your vegetables. Link to YouTube<br><br>Scene 3. 1:57 – Loop of scene 0. Your credit card is declined. Person in line is increasingly impatient and upset. Link to YouTube | 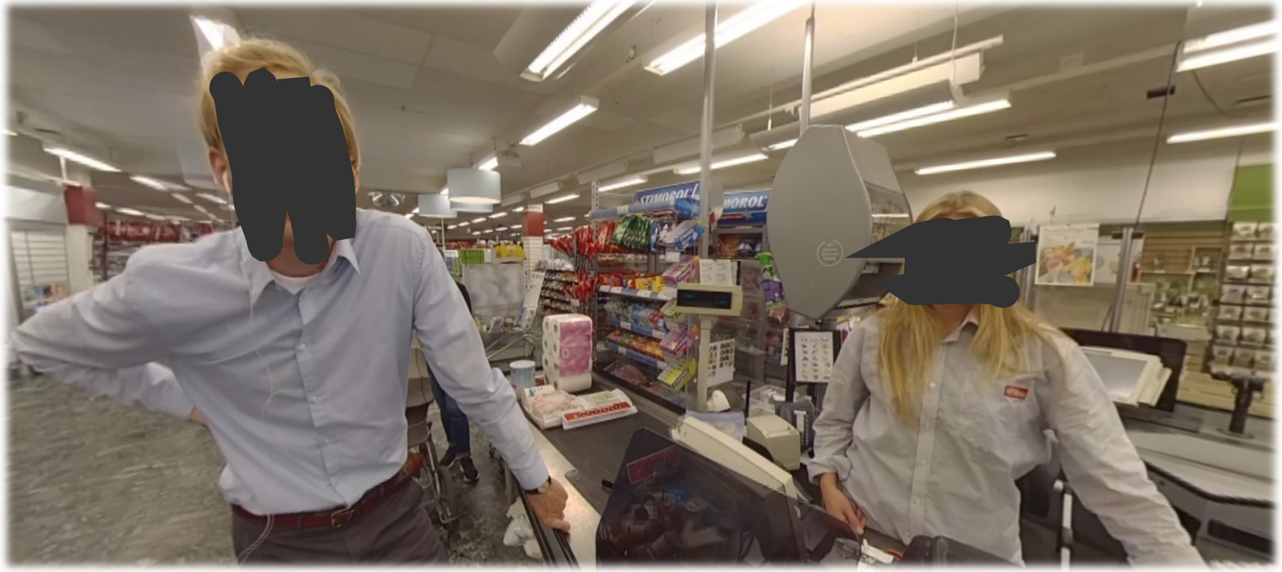 |

| | |
|---|---|
| Scene 4. 0:50 – Loop of scene 0. You win a prize for being customer number 1.000.000. Link to YouTube | |
| **Environment 2 – Presentation**<br>Scene 0. Loop – Standing in meeting room alone. Link to YouTube<br><br>Scene 1. 2:20 – Loop of scene 0. Meeting preparations with colleague. Link to YouTube<br><br>Scene 2. 3:29 – Loop of scene 0. Contact person arrives. Short conversation. Link to YouTube<br><br>Scene 3. 2:35 – Loop of scene 0. Two important meeting participants arrive. Link to YouTube | 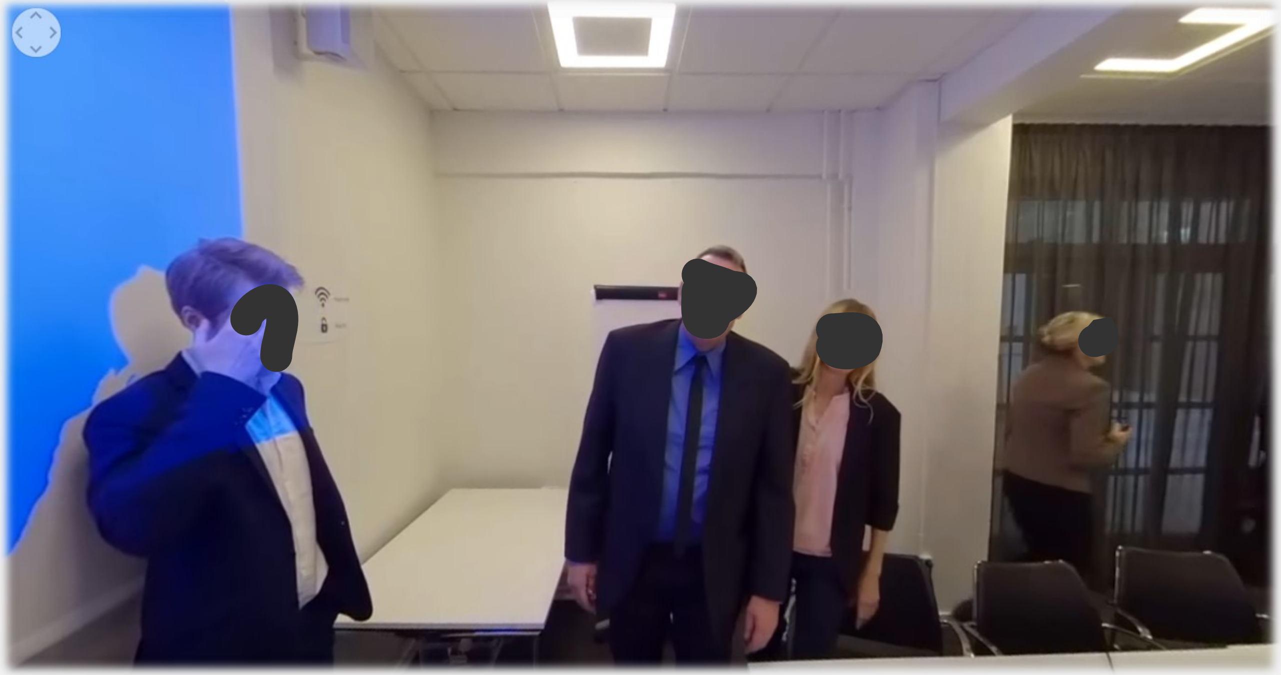 |

| | |
|---|---|
| Scene 4. 2:01 – Loop of scene 0. The rest of the meeting participants arrive. Introductions to the group. Link to YouTube<br><br>Scene 5. 4:02 – Loop of scene 0. Presentation has technical difficulties. Partner leaves mid-presentation. Link to YouTube<br><br>Scene 6. 2:07 – Loop of scene 0. Scolding from the boss. Link to YouTube | |

**Environment 3 – Cafeteria**

Scene 0. Loop – Sitting by table. One person sits down nearby. Link to YouTube

Scene 1. 3:04 – Loop of scene 0. Someone small talks near you. You are asked about parking. Few people in the room. Link to YouTube

Scene 2. 4:30 – Loop of scene 0. More small talk. You are asked if there is room by the table. Link to YouTube

Scene 3. 5:15 – Loop of scene 0. You are in the middle of a discussion about art. Link to YouTube

Scene 4. 5:11 – Loop of scene 0. You are in the middle of a

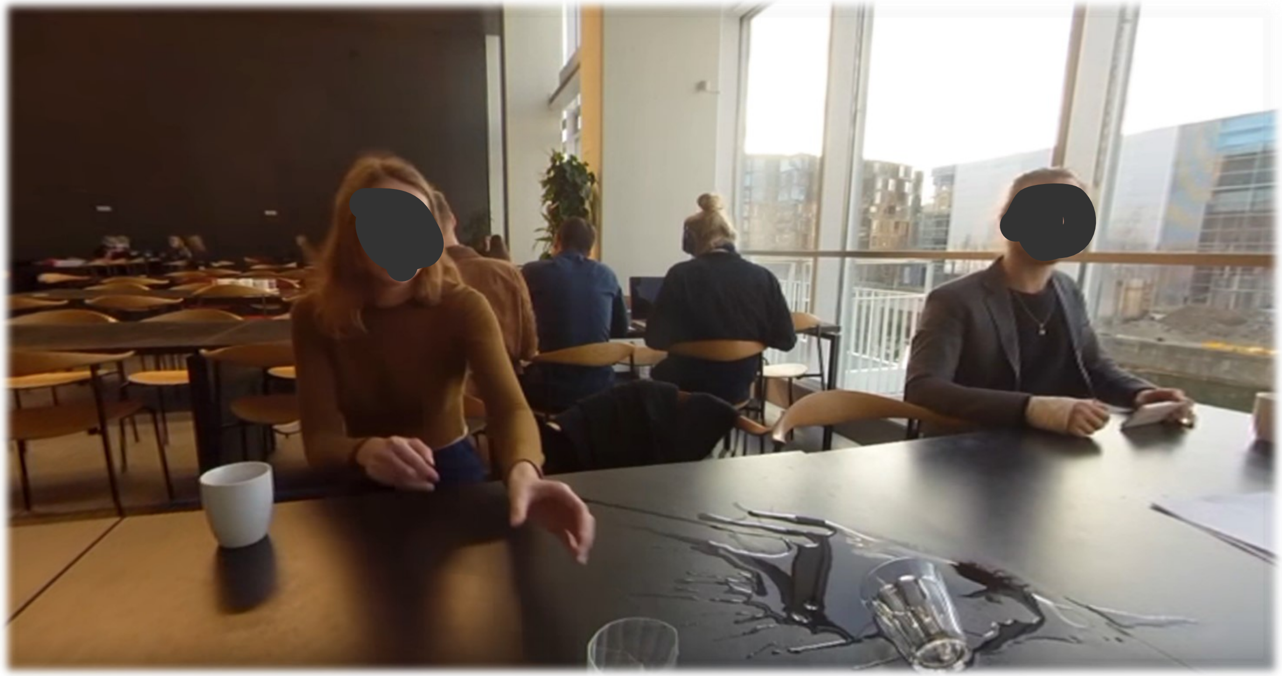

| | |
|---|---|
| heated discussion about transgender issues. Link to YouTube | |
| **Environment 4 – Party**<br>Scene 0. Loop – Arrived at door. Party audible inside.<br><br>Scene 1. 1:19 – Loop of scene 0. Guest arrives. Host opens door and greets guest. Link to YouTube<br><br>Scene 2. Loop – In kitchen with many partygoers. You are offered a shot of an alcoholic beverage. Link to YouTube<br><br>Scene 3. 3:35 – Loop of scene 2. Participate in drinking game in the kitchen. Link to YouTube<br><br>Scene 4. 3:37 – Loop of scene 2. In corner of room. Two guests | 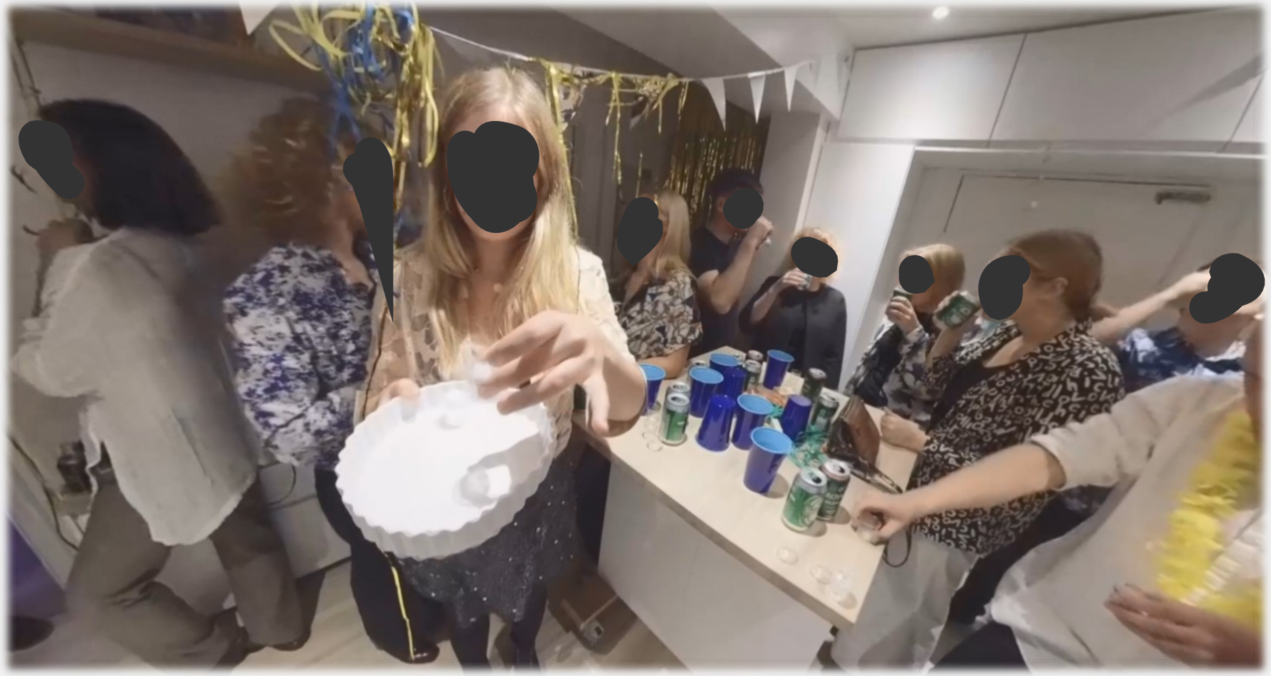 |

| | |
|---|---|
| have an intimate conversation close by. Link to YouTube<br><br>Scene 5. 2:54 – Loop of scene 2. On the dancefloor. A circle of dancing revelers forms around you. Link to YouTube | |
| **Environment 5 – Auditorium**<br>Scene 0A. Loop. Sitting at a lecture. Link to YouTube<br>Scene 0B. Loop. Waiting for lecture to start. Few other people. Link to YouTube<br><br>Scene 1. 1:14 – Loop of scene 0A. Arrived before class start to empty auditorium. Link to YouTube<br><br>Scene 2. 0:49 – Loop of scene 0A. Arrived exactly at the right time. Few people in the auditorium. Link to YouTube | 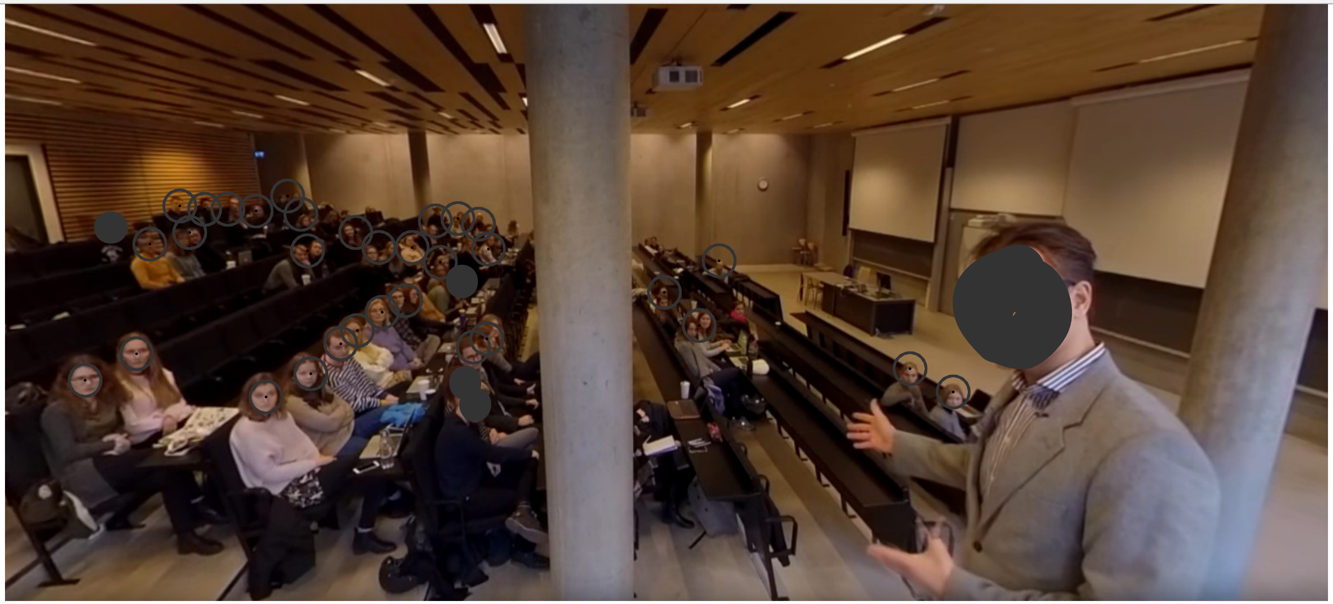 |

| | |
|---|---|
| Scene 3. 1:02 – Loop of scene 0A. Arrived too late. Professor notes it as you enter.<br>Link to YouTube<br><br>Scene 4. 1:18 – Loop of scene 0A. Arrived much too late. Scolded in front of full auditorium by professor.<br>Link to YouTube | |

| **Environment 6 – Job interview** | |
|---|---|
| A variety of relevant questions to be posed can be chosen by the patient such as "What are your weaknesses" etc., after the question a "listening loop" is played that allows the patient to talk while the two interviewers appear to listen. | 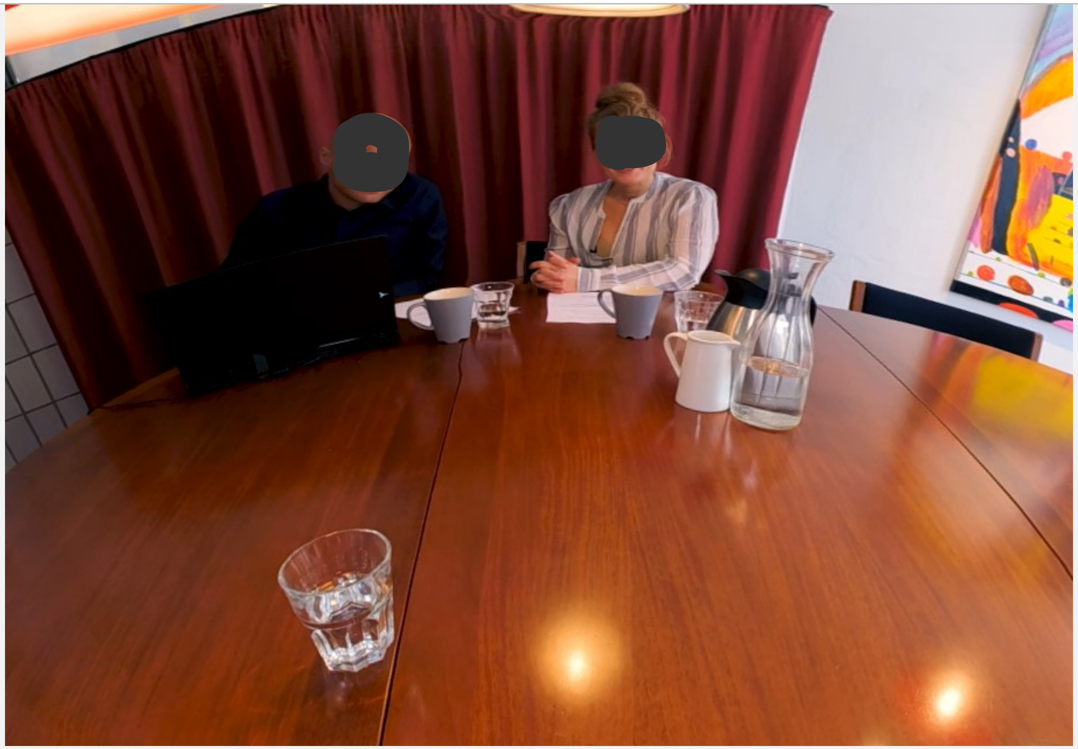 |

**Environment 7 – Crossing a bridge**

Scene 0A. Loop. Waiting in a highway rest area.

Scene 0B. Loop. Waiting to get picked up in sub-urban area.

Scene 1. 1:38 – Loop of scene 2. Driving in sub-urban area. Picking up other passengers.

Scene 2. Loop. Driving, no conversation.

Scene 3. Loop. Crossing a bridge, no conversation.

Scene 4. 4:25 -- Loop of scene 2. Passenger gets carsick.

Scene 5. 5:27. Car breaks down while crossing bridge.

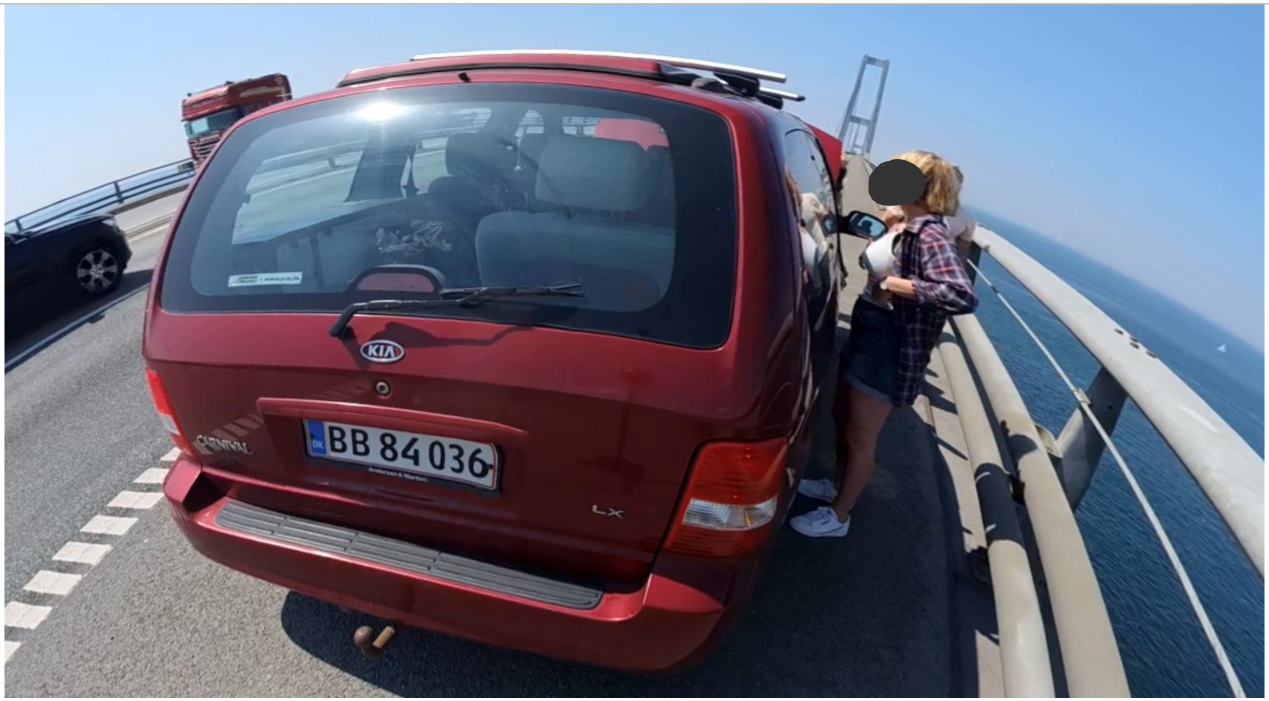

**Environment 8 - Small talking/discussing in a canteen in a work setting**

Scene 0. Loop. At the buffet.

Scene 1. 1:00 – Loop of Scene 0. Standing in line.

Scene 2. 3:00 – Loop of Scene 0. In the middle of the canteen.

Scene 3. Loop.  Eating with colleagues. Small talk.

Scene 4. 2:00 – Loop of Scene 3. Standing by table. Positive mood.

Scene 5. 5:40 – Loop of Scene 3 Eating with colleagues. Negative mood.

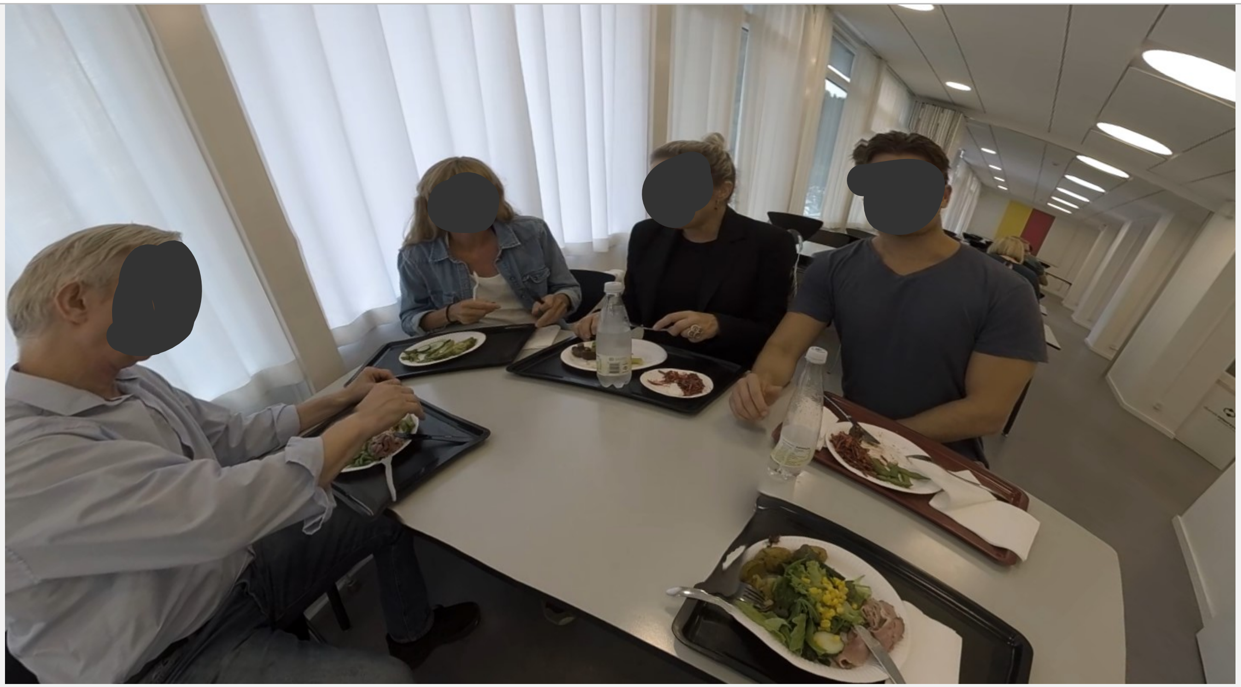

| | |
|---|---|
| Scene 6. 2:00 – Loop of Scene 0. Drops tray with food next to table. | |
| **Environment 9 - Taking a commercial airplane**<br><br>Scene 0. 7:26. Taking a plane, from boarding to landing.<br><br>It is possible to only play specific segments, e.g. “Turbulence” or “Boarding”. | 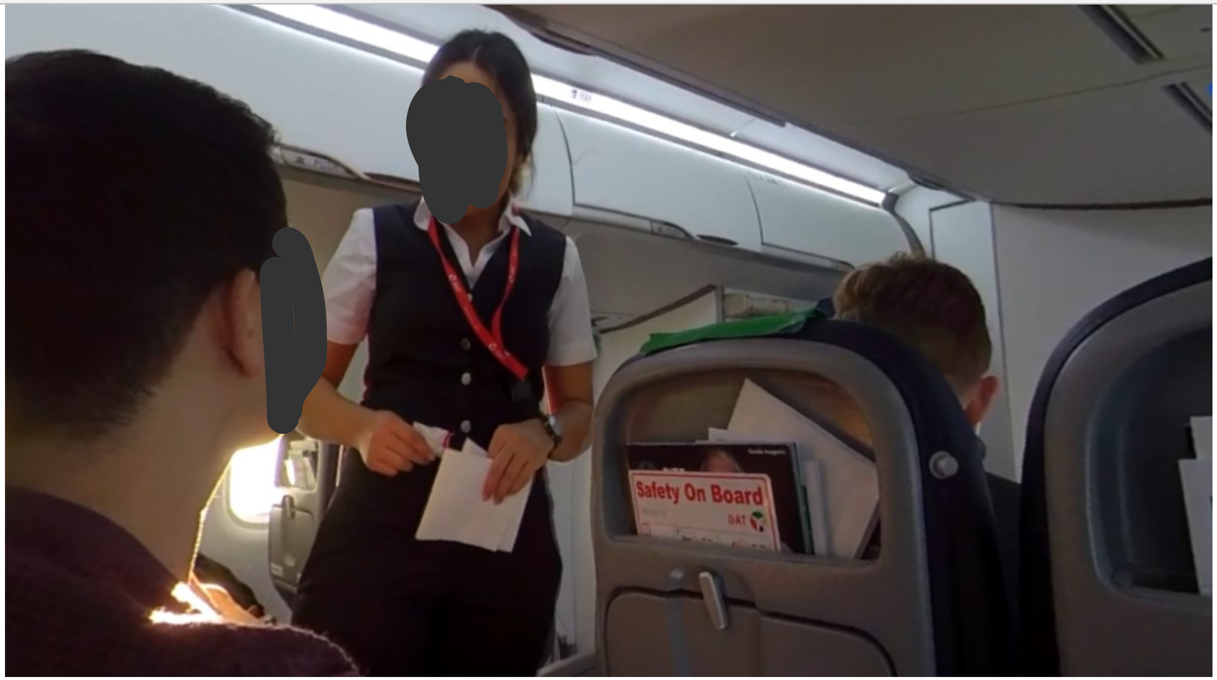 |

| | |
|---|---|
| **Environment 10 - Being in a crowded shopping center**<br><br>Scene 0. Loop. At entrance to mall.<br><br>Scene 1. Loop. Inside mall, not crowded.<br><br>Scene 2. Loop. Inside mall, crowded.<br><br>Scene 4. Loop. Standing in line to toilet. One is out of order. | 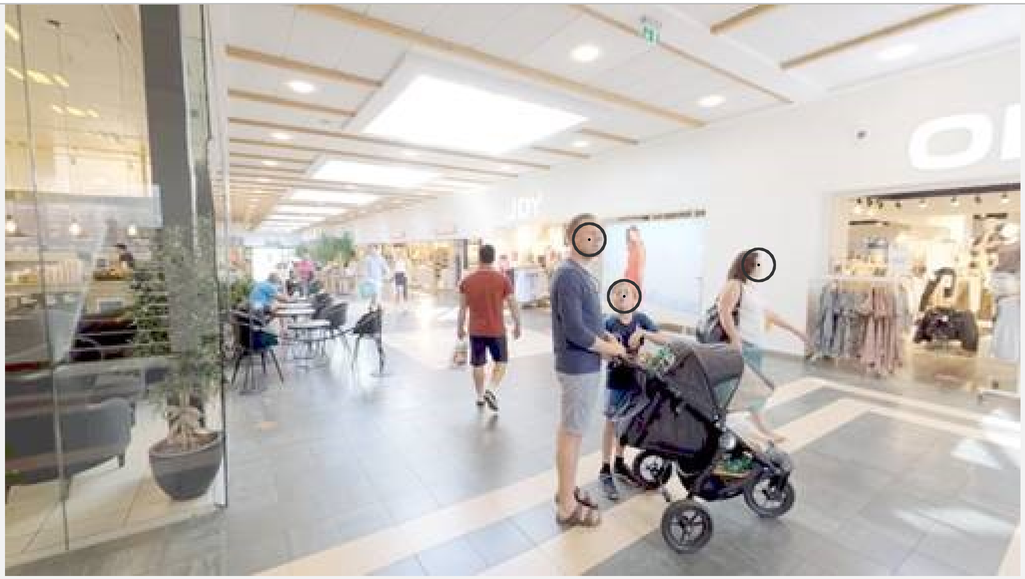 |

Environment 11 - Taking an elevator

Scene 0. Loop. Waiting for elevator.

Scene 1. Loop. Taking the elevator alone.

Scene 2. 1:45 – Loop of Scene 1. Taking the elevator with other people.

Scene 3. 6:30 – Loop of Scene 1. Elevator malfunctions with other people.

Scene 4. 6:20 – Loop of Scene 1 Elevator malfunctions with other people. You have a panic attack.

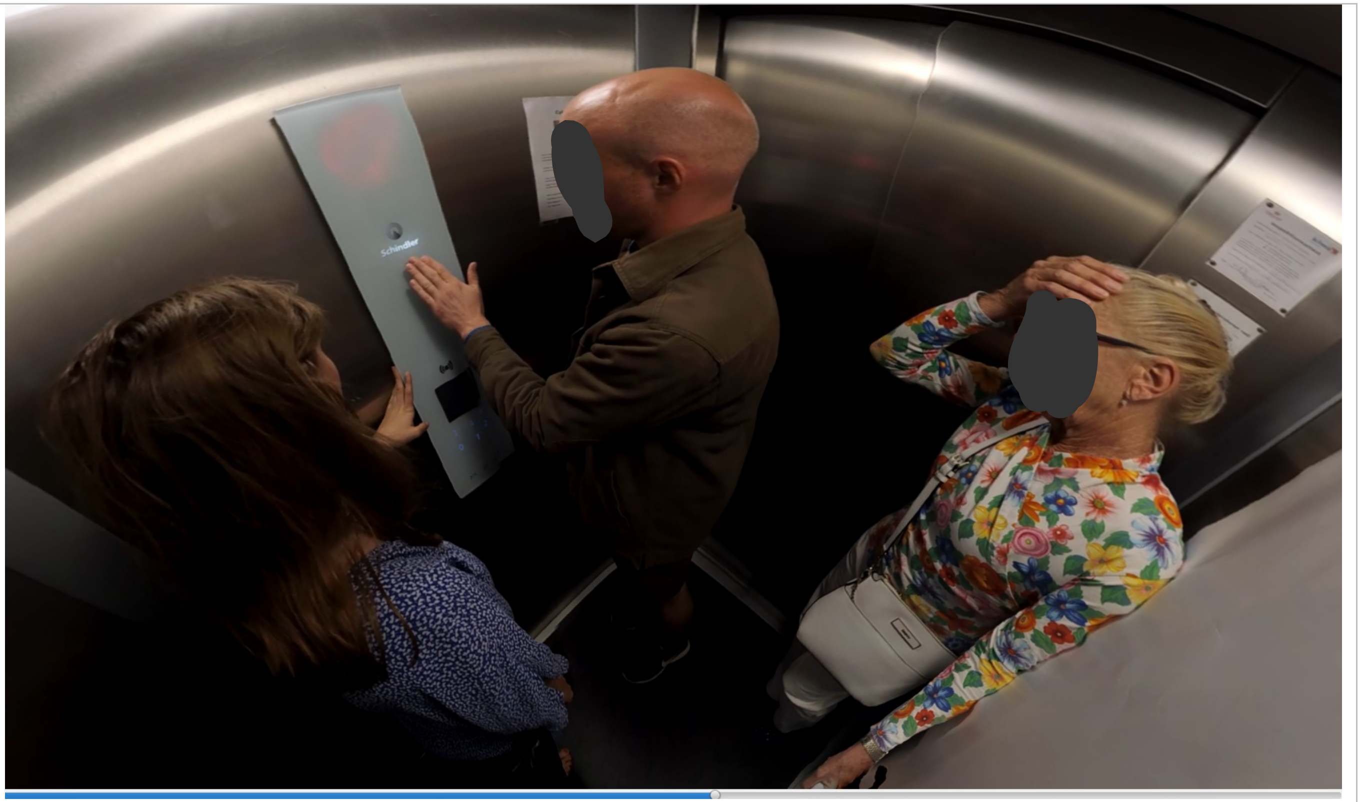

Environment 12 - Waiting for- and taking the bus

Scene 0. Loop. Waiting for bus.

Scene 1. 0:50 – Loop of Scene 2A. Bus arrives. Entering bus.

Scene 2A. Loop. In driving bus, sitting.

Scene 2B. Loop. In driving bus, standing.

Scene 3. 0:50 – Loop of Scene 2A. Baby driving in bus.

Scene 4. 2:00 – Loop of Scene 2A. Man speaks loudly on the phone next to you.

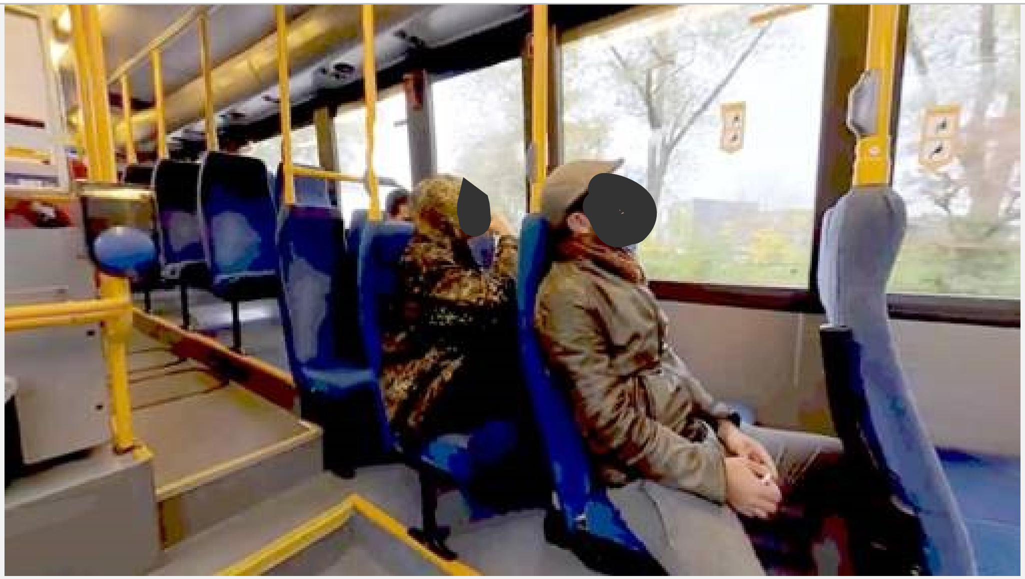

| | |
|---|---|
| Scene 5. 3:30 – Loop of Scene 2A. Drunk man enters bus and addresses you.<br><br>Scene 6. 1:20 – Loop of Scene 2A. Elderly lady asks for your seat. You refuse.<br><br>Scene 7. 2:00 – Loop of Scene 2A. Baby cries, man speaks loudly on phone and drunk man addresses you. | |

| | |
|---|---|
| Environment 13 - Leaving your apartment<br><br>Scene 0. Loop. In entrance of apartment.<br><br>Scene 1. Loop. On apartment staircase outside apartment.<br><br>Scene 2. Loop. Standing in the entrance to the apartment building.<br><br>Scene 3. Loop. Standing in the street outside apartment. | 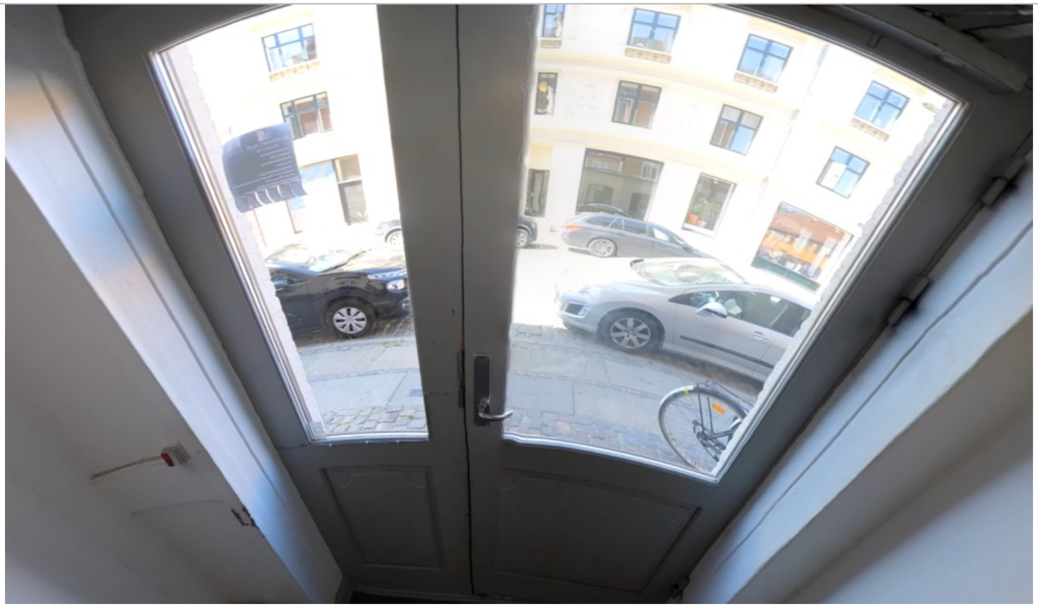 |

*"—Loop of 0/1/2" indicates that the scene automatically jumps to that loop after finishing*
