# Supplementary material for: Group Cognitive Behavioral Therapy With Virtual Reality Exposure Versus In-Vivo Exposure for Social Anxiety Disorder and Agoraphobia: Underpowered Results From the SoREAL Pragmatic Randomized Clinical Trial
Source: JMIR Ment Health. 2025 Nov 3;12:e73815. doi: 10.2196/73815 (PMC12582524; doi:10.2196/73815)
Supplement: Multimedia Appendix 2 [file mental-v12-e73815-s002.pdf]

Multimedia Appendix 2: Post Hoc Analyses

Table 6. ANCOVAs of between-group differences in change scores from baseline to post-treatment and 1-year follow-up.

Outcome measure	Δ Baseline → Post						Δ Baseline → Follow-up					
	Mean diff.	95% CI	SE	F	p	d	Mean diff.	95% CI	SE	F	p	d
Phobic anxiety severity (LSAS and MIA POMP transformed)	1.236	-4.540 to 7.012	2.91	0.180	.67	0.07	2.319	-3.356 to 7.995	2.89	0.610	.44	0.12
Fear of negative evaluation (FNES)	0.675	-4.015 to 5.366	2.33	0.083	.77	0.10	-0.493	-5.171 to 4.184	2.36	0.032	.86	-0.07
Depressive symptoms (HAM-6)	0.280	-1.475 to 2.035	0.87	0.101	.75	0.07	0.207	-1.698 to 2.111	0.95	0.047	.83	0.05
Functioning (WSAS)	0.311	-0.630 to 1.252	0.47	0.434	.51	0.12	0.019	-1.007 to 1.045	0.51	0.001	.97	0.01
Quality of life (WHO-5)	-1.401	-7.494 to 4.693	3.06	0.210	.65	-0.07	-0.733	-6.826 to 5.361	3.08	0.050	.82	-0.03
Self-belief of coping (GSE)	-0.189	-1.691 to 1.313	0.75	0.059	.81	-0.04	-0.152	-1.687 to 1.384	0.76	0.020	.89	-0.03
Alcohol use (TLFB, units last 30 days)	0.215	-0.173 to 0.788	0.19	0.993	.32	0.02	0.154	-0.221 to 0.711	0.2	0.714	.48	0.01
Social functioning (PSP)	0.738	-1.398 to 2.875	1.07	0.473	.49	0.11	-0.041	-2.356 to 2.274	1.15	0.001	.97	-0.01

Data is pooled from 100 imputations. Negative values indicate a greater reduction or improvement in the VR-CBT group compared to the CBT group. Covariates are primary diagnoses and comorbidity. Phobic anxiety severity included sessions attended as covariate.

LSAS=Leibowitz Social Anxiety Scale. MIA=Mobility Inventory for Agoraphobia. FNES=Fear of Negative Evaluation Scale. HAM-6=Hamilton Depression Scale. WHO-5=World Health Organization Wellbeing Index. WSAS=Work and Social Adjustment Scale. GSE=General Self-Efficacy Scale. PSP=Personal and Social Performance Scale. TLFB=Timeline Followback for alcohol consumption.

Table 7. Multilevel models of group differences for primary and secondary symptom, quality of life and functioning outcomes

Data are pooled from 100 imputations. Negative values indicate greater improvement or symptom reduction in the VR-CBT group compared to the CBT group. All models adjust for primary diagnosis and comorbidity; the model for phobic anxiety severity additionally adjusts for number of sessions attended.

Outcome measure	Post-treatment		1-year follow-up	
	B (SE) [95% CI]	p	B (SE) [95% CI]	p
Phobic anxiety severity (LSAS and MIA POMP transformed)	-1.302 (2.845) [–6.881, 4.277]	.647	-2.287 (2.579) [–7.347, 2.772]	.375
Fear of negative evaluation (FNES)	-0.091 (0.152) [–0.389, 0.207]	.550	-0.076 (0.154) [–0.379, 0.227]	.623
Depressive symptoms (HAM-6)	-0.472 (0.841) [–2.121, 1.178]	.575	0.035 (0.840) [–1.613, 1.683]	.967
Functioning (WSAS)	-0.005 (0.386) [–0.762, 0.753]	.990	-0.168 (0.411) [–0.975, 0.638]	.682
Wellbeing (WHO-5)	-1.470 (4.048) [–9.411, 6.471]	.717	1.468 (4.570) [–7.501, 10.436]	.748

LSAS=Liebowitz Social Anxiety Scale. MIA=Mobility Inventory for Agoraphobia. FNES=Fear of Negative Evaluation Scale. HAM-6=Hamilton Depression Scale. WSAS=Work and Social Adjustment Scale. WHO-5=World Health Organization Wellbeing Index.

Table 8. ANCOVA with random forest imputation for primary outcome and FNES.

ANCOVA models included primary diagnosis and comorbidity as covariates. The model for phobic anxiety severity additionally included the number of sessions attended. Negative B values indicate greater improvement in the VR-CBT group compared to the CBT group.

Outcome Measure	Post-treatment					Follow-up				
	B	95 % CI	SE	P	Cohen's D	B	95 % CI	SE	P	Cohen's D
Phobic anxiety severity (LSAS and MIA POMP transformed)	–1.081	–6.000, 3.838	2.490	0.66	–0.055	–0.112	–2.974, 2.750	1.447	0.93	–0.006
Fear of negative evaluation (FNES)	–0.640	–0.342, 0.213	0.142	0.65	–0.061	0.047	–0.215, 0.309	0.352	0.730	0.039

LSAS=Liebowitz Social Anxiety Scale. MIA=Mobility Inventory for Agoraphobia. FNES=Fear of Negative Evaluation Scale.
