# Supplementary material for: Group Cognitive Behavioral Therapy With Virtual Reality Exposure Versus In-Vivo Exposure for Social Anxiety Disorder and Agoraphobia: Underpowered Results From the SoREAL Pragmatic Randomized Clinical Trial
Source: JMIR Ment Health. 2025 Nov 3;12:e73815. doi: 10.2196/73815 (PMC12582524; doi:10.2196/73815)
Supplement: Checklist 1 [file mental-v12-e73815-s003.pdf]

CONSORT-EHEALTH (V 1.6.1) - Submission/Publication Form

The CONSORT-EHEALTH checklist is intended for authors of randomized trials evaluating web-based and Internet-based applications/interventions, including mobile interventions, electronic games (incl multiplayer games), social media, certain telehealth applications, and other interactive and/or networked electronic applications. Some of the items (e.g. all subitems under item 5 - description of the intervention) may also be applicable for other study designs.

The goal of the CONSORT EHEALTH checklist and guideline is to be
a) a guide for reporting for authors of RCTs,
b) to form a basis for appraisal of an ehealth trial (in terms of validity)

CONSORT-EHEALTH items/subitems are MANDATORY reporting items for studies published in the Journal of Medical Internet Research and other journals / scientific societies endorsing the checklist.

Items numbered 1., 2., 3., 4a., 4b etc are original CONSORT or CONSORT-NPT (non-pharmacologic treatment) items.
Items with Roman numerals (i., ii, iii, iv etc.) are CONSORT-EHEALTH extensions/clarifications.

As the CONSORT-EHEALTH checklist is still considered in a formative stage, we would ask that you also RATE ON A SCALE OF 1-5 how important/useful you feel each item is FOR THE PURPOSE OF THE CHECKLIST and reporting guideline (optional).

Mandatory reporting items are marked with a red *.
In the textboxes, either copy & paste the relevant sections from your manuscript into this form - please include any quotes from your manuscript in QUOTATION MARKS,
or answer directly by providing additional information not in the manuscript, or elaborating on why the item was not relevant for this study.

YOUR ANSWERS WILL BE PUBLISHED AS A SUPPLEMENTARY FILE TO YOUR PUBLICATION IN JMIR AND ARE CONSIDERED PART OF YOUR PUBLICATION (IF ACCEPTED).
Please fill in these questions diligently. Information will not be copyedited, so please use proper spelling and grammar, use correct capitalization, and avoid abbreviations.

DO NOT FORGET TO SAVE AS PDF _AND_ CLICK THE SUBMIT BUTTON SO YOUR ANSWERS ARE IN OUR DATABASE !!!

Citation Suggestion (if you append the pdf as Appendix we suggest to cite this paper in the caption):
Eysenbach G, CONSORT-EHEALTH Group
CONSORT-EHEALTH: Improving and Standardizing Evaluation Reports of Web-based and Mobile Health Interventions
J Med Internet Res 2011;13(4):e126

URL: http://www.jmir.org/2011/4/e126/
doi: 10.2196/jmir.1923
PMID: 22209829

benjamin.ata@gmail.com Switch account

Not shared

Draft saved

* Indicates required question

Your name *

First Last

Benjamin Alexander Thorup Arnfred

Primary Affiliation (short), City, Country *

University of Toronto, Toronto, Canada

Copenhagen University Hospital, Copenhagen,

Your e-mail address *

abc@gmail.com

barn0006@regionh.dk

Title of your manuscript *

Provide the (draft) title of your manuscript.

Underpowered results from the SoREAL Randomized Clinical Trial: Group Cognitive Behavioral Therapy with Virtual Reality Exposure Versus In-Vivo Exposure for Social Anxiety Disorder and Agoraphobia

Name of your App/Software/Intervention *

If there is a short and a long/alternate name, write the short name first and add the long name in brackets.

SoREAL Exposure

Evaluated Version (if any)

e.g. "V1", "Release 2017-03-01", "Version 2.0.27913"

V1.00

Language(s) *

What language is the intervention/app in? If multiple languages are available, separate by comma (e.g. "English, French")

Danish

URL of your Intervention Website or App

e.g. a direct link to the mobile app on app in appstore (itunes, Google Play), or URL of the website. If the intervention is a DVD or hardware, you can also link to an Amazon page.

Your answer

URL of an image/screenshot (optional)

Your answer

Accessibility *

Can an enduser access the intervention presently?

- ○ access is free and open
- ○ access only for special usergroups, not open
- ◉ access is open to everyone, but requires payment/subscription/in-app purchases
- ○ app/intervention no longer accessible
- ○ Other:

Primary Medical Indication/Disease/Condition *

e.g. "Stress", "Diabetes", or define the target group in brackets after the condition, e.g. "Autism (Parents of children with)", "Alzheimers (Informal Caregivers of)"

Social phobia and agoraphobia

Primary Outcomes measured in trial *

comma-separated list of primary outcomes reported in the trial

Liebowitz Social Anxiety Scale, Mobility Invent

Secondary/other outcomes

Are there any other outcomes the intervention is expected to affect?

Fear of negative evaluation, measured with the Brief Version of the Fear of Negative Evaluation Scale]; depression symptoms, measured with the six-item version of the Hamilton Depression Rating Scale; work and social functioning, measured with the Work and Social Adjustment Scale; treatment satisfaction, measured with the Client Satisfaction Questionnaire; and quality of life, measured with the WHO-5 Well-Being Index.

Recommended "Dose" *

What do the instructions for users say on how often the app should be used?

- ( ) Approximately Daily
- (x) Approximately Weekly
- ( ) Approximately Monthly
- ( ) Approximately Yearly
- ( ) "as needed"
- ( ) Other:

Approx. Percentage of Users (starters) still using the app as recommended after 3 months *

- ( ) unknown / not evaluated
- ( ) 0-10%
- ( ) 11-20%
- ( ) 21-30%
- ( ) 31-40%
- ( ) 41-50%
- ( ) 51-60%
- ( ) 61-70%
- ( ) 71%-80%
- ( ) 81-90%
- ( ) 91-100%
- (x) Other: Not relevant. Patients lose access to app after 3 month.

Overall, was the app/intervention effective? *

- ○ yes: all primary outcomes were significantly better in intervention group vs control
- ○ partly: SOME primary outcomes were significantly better in intervention group vs control
- ○ no statistically significant difference between control and intervention
- ○ potentially harmful: control was significantly better than intervention in one or more outcomes
- ◉ inconclusive: more research is needed
- ○ Other:

Article Preparation Status/Stage *

At which stage in your article preparation are you currently (at the time you fill in this form)

- ○ not submitted yet - in early draft status
- ○ not submitted yet - in late draft status, just before submission
- ◉ submitted to a journal but not reviewed yet
- ○ submitted to a journal and after receiving initial reviewer comments
- ○ submitted to a journal and accepted, but not published yet
- ○ published
- ○ Other:

Journal *

If you already know where you will submit this paper (or if it is already submitted), please provide the journal name (if it is not JMIR, provide the journal name under "other")

- ◯ not submitted yet / unclear where I will submit this
- ◯ Journal of Medical Internet Research (JMIR)
- ◯ JMIR mHealth and UHealth
- ◯ JMIR Serious Games
- ◉ JMIR Mental Health
- ◯ JMIR Public Health
- ◯ JMIR Formative Research
- ◯ Other JMIR sister journal
- ◯ Other:

Is this a full powered effectiveness trial or a pilot/feasibility trial? *

- ◯ Pilot/feasibility
- ◉ Fully powered

Manuscript tracking number *

If this is a JMIR submission, please provide the manuscript tracking number under "other" (The ms tracking number can be found in the submission acknowledgement email, or when you login as author in JMIR. If the paper is already published in JMIR, then the ms tracking number is the four-digit number at the end of the DOI, to be found at the bottom of each published article in JMIR)

- ◯ no ms number (yet) / not (yet) submitted to / published in JMIR
- ◉ Other: 73815

TITLE AND ABSTRACT

1a) TITLE: Identification as a randomized trial in the title

1a) Does your paper address CONSORT item 1a? *

I.e does the title contain the phrase "Randomized Controlled Trial"? (if not, explain the reason under "other")

- (x) yes
- ( ) Other:

1a-i) Identify the mode of delivery in the title

Identify the mode of delivery. Preferably use “web-based” and/or “mobile” and/or “electronic game” in the title. Avoid ambiguous terms like “online”, “virtual”, “interactive”. Use “Internet-based” only if Intervention includes non-web-based Internet components (e.g. email), use “computer-based” or “electronic” only if offline products are used. Use “virtual” only in the context of “virtual reality” (3-D worlds). Use “online” only in the context of “online support groups”. Complement or substitute product names with broader terms for the class of products (such as “mobile” or “smart phone” instead of “iphone”), especially if the application runs on different platforms.

	1	2	3	4	5	
subitem not at all important	( )	( )	( )	( )	(x)	essential

Clear selection

Does your paper address subitem 1a-i? *

Copy and paste relevant sections from manuscript title (include quotes in quotation marks "like this" to indicate direct quotes from your manuscript), or elaborate on this item by providing additional information not in the ms, or briefly explain why the item is not applicable/relevant for your study

"Group Cognitive Behavioral Therapy with Virtual Reality Exposure"

1a-ii) Non-web-based components or important co-interventions in title

Mention non-web-based components or important co-interventions in title, if any (e.g., “with telephone support”).

	1	2	3	4	5	
subitem not at all important	○	○	○	○	◉	essential

Clear selection

Does your paper address subitem 1a-ii?

Copy and paste relevant sections from manuscript title (include quotes in quotation marks "like this" to indicate direct quotes from your manuscript), or elaborate on this item by providing additional information not in the ms, or briefly explain why the item is not applicable/relevant for your study

"Group Cognitive Behavioral Therapy"

1a-iii) Primary condition or target group in the title

Mention primary condition or target group in the title, if any (e.g., “for children with Type I Diabetes”) Example: A Web-based and Mobile Intervention with Telephone Support for Children with Type I Diabetes: Randomized Controlled Trial

	1	2	3	4	5	
subitem not at all important	○	○	○	○	◉	essential

Clear selection

Does your paper address subitem 1a-iii? *

Copy and paste relevant sections from manuscript title (include quotes in quotation marks "like this" to indicate direct quotes from your manuscript), or elaborate on this item by providing additional information not in the ms, or briefly explain why the item is not applicable/relevant for your study

"for Social Anxiety Disorder and Agoraphobia"

1b) ABSTRACT: Structured summary of trial design, methods, results, and conclusions

NPT extension: Description of experimental treatment, comparator, care providers, centers, and blinding status.

1b-i) Key features/functionalities/components of the intervention and comparator in the METHODS section of the ABSTRACT

Mention key features/functionalities/components of the intervention and comparator in the abstract. If possible, also mention theories and principles used for designing the site. Keep in mind the needs of systematic reviewers and indexers by including important synonyms. (Note: Only report in the abstract what the main paper is reporting. If this information is missing from the main body of text, consider adding it)

	1	2	3	4	5	
subitem not at all important	○	○	○	○	◉	essential

Clear selection

Does your paper address subitem 1b-i? *

Copy and paste relevant sections from the manuscript abstract (include quotes in quotation marks "like this" to indicate direct quotes from your manuscript), or elaborate on this item by providing additional information not in the ms, or briefly explain why the item is not applicable/relevant for your study

"In this randomized, parallel-group, assessor-blinded trial, 177 participants with SAD or agoraphobia were assigned to either VR-CBT or traditional CBT across five Danish mental health outpatient clinics. Both groups received 14 weekly group sessions."

1b-ii) Level of human involvement in the METHODS section of the ABSTRACT

Clarify the level of human involvement in the abstract, e.g., use phrases like “fully automated” vs. “therapist/nurse/care provider/physician-assisted” (mention number and expertise of providers involved, if any). (Note: Only report in the abstract what the main paper is reporting. If this information is missing from the main body of text, consider adding it)

	1	2	3	4	5	
subitem not at all important	◉	○	○	○	○	essential

Clear selection

Does your paper address subitem 1b-ii?

Copy and paste relevant sections from the manuscript abstract (include quotes in quotation marks "like this" to indicate direct quotes from your manuscript), or elaborate on this item by providing additional information not in the ms, or briefly explain why the item is not applicable/relevant for your study

This is not typically reported in the abstract of psychotherapy RCTs. However, it is described in the main text.

1b-iii) Open vs. closed, web-based (self-assessment) vs. face-to-face assessments in the METHODS section of the ABSTRACT

Mention how participants were recruited (online vs. offline), e.g., from an open access website or from a clinic or a closed online user group (closed usergroup trial), and clarify if this was a purely web-based trial, or there were face-to-face components (as part of the intervention or for assessment). Clearly say if outcomes were self-assessed through questionnaires (as common in web-based trials). Note: In traditional offline trials, an open trial (open-label trial) is a type of clinical trial in which both the researchers and participants know which treatment is being administered. To avoid confusion, use “blinded” or “unblinded” to indicated the level of blinding instead of “open”, as “open” in web-based trials usually refers to “open access” (i.e. participants can self-enrol). (Note: Only report in the abstract what the main paper is reporting. If this information is missing from the main body of text, consider adding it)

	1	2	3	4	5	
subitem not at all important	○	○	◉	○	○	essential

Clear selection

Does your paper address subitem 1b-iii?

Copy and paste relevant sections from the manuscript abstract (include quotes in quotation marks "like this" to indicate direct quotes from your manuscript), or elaborate on this item by providing additional information not in the ms, or briefly explain why the item is not applicable/relevant for your study

"assessor-blinded trial". We do not state how patients were recruited in the abstract, but we describe it in the main text.

1b-iv) RESULTS section in abstract must contain use data

Report number of participants enrolled/assessed in each group, the use/uptake of the intervention (e.g., attrition/adherence metrics, use over time, number of logins etc.), in addition to primary/secondary outcomes. (Note: Only report in the abstract what the main paper is reporting. If this information is missing from the main body of text, consider adding it)

	1	2	3	4	5	
subitem not at all important	○	○	◉	○	○	essential

Clear selection

Does your paper address subitem 1b-iv?

Copy and paste relevant sections from the manuscript abstract (include quotes in quotation marks "like this" to indicate direct quotes from your manuscript), or elaborate on this item by providing additional information not in the ms, or briefly explain why the item is not applicable/relevant for your study

"177 participants with SAD or agoraphobia were assigned to either VR-CBT (N=81) or traditional CBT (N=96)". We do not report numerical results directly in the abstract due to the underpowered nature of the trial. We judge that the context of the trial is nessecary to evaluate the concrete results.

1b-v) CONCLUSIONS/DISCUSSION in abstract for negative trials

Conclusions/Discussions in abstract for negative trials: Discuss the primary outcome - if the trial is negative (primary outcome not changed), and the intervention was not used, discuss whether negative results are attributable to lack of uptake and discuss reasons. (Note: Only report in the abstract what the main paper is reporting. If this information is missing from the main body of text, consider adding it)

	1	2	3	4	5	
subitem not at all important	○	○	○	○	◉	essential

Clear selection

Does your paper address subitem 1b-v?

Copy and paste relevant sections from the manuscript abstract (include quotes in quotation marks "like this" to indicate direct quotes from your manuscript), or elaborate on this item by providing additional information not in the ms, or briefly explain why the item is not applicable/relevant for your study

"Due to insufficient recruitment and substantial missing data, no definitive conclusions can be drawn regarding group differences between VR-CBT and traditional CBT in group settings. The feasibility issues encountered suggest that careful consideration of the benefits and limitations of VR technology is essential before implementation in clinical practice."

INTRODUCTION

2a) In INTRODUCTION: Scientific background and explanation of rationale

2a-i) Problem and the type of system/solution

Describe the problem and the type of system/solution that is object of the study: intended as stand-alone intervention vs. incorporated in broader health care program? Intended for a particular patient population? Goals of the intervention, e.g., being more cost-effective to other interventions, replace or complement other solutions? (Note: Details about the intervention are provided in "Methods" under 5)

	1	2	3	4	5	
subitem not at all important	○	○	○	○	●	essential

Clear selection

Does your paper address subitem 2a-i? *

Copy and paste relevant sections from the manuscript (include quotes in quotation marks "like this" to indicate direct quotes from your manuscript), or elaborate on this item by providing additional information not in the ms, or briefly explain why the item is not applicable/relevant for your study

"Social anxiety disorder (SAD) and panic disorder with or without agoraphobia (PD/A) are prevalent [1,2] and impairing disorders associated with decreased quality of life [3], increased suicidality [4], and psychiatric comorbidity. They typically develop in early adolescence [1,2], are more common in women [5,6], and are often chronic if untreated [7,8].

Cognitive behavioral therapy (CBT) is a research-informed, time-limited psychotherapeutic intervention focused on modifying biased cognitions and dysfunctional behaviors [9,10]. It is the recommended first-line treatment for SAD and PD/A [11–14], demonstrating lasting efficacy [15–21], minimal side effects [15], and is generally preferred over psychopharmacological treatment [22]. Exposure therapy, a key component of CBT for SAD and PD/A, aims to modify patients' expectations about the likelihood and consequences of a feared outcome by exposing them to feared stimuli [23,24]. However, feared stimuli can sometimes be inaccessible, uncontrollable, or aversive, leading both patients and therapists to avoid exposure therapy [25–28]."

2a-ii) Scientific background, rationale: What is known about the (type of) system

Scientific background, rationale: What is known about the (type of) system that is the object of the study (be sure to discuss the use of similar systems for other conditions/diagnoses, if appropiate), motivation for the study, i.e. what are the reasons for and what is the context for this specific study, from which stakeholder viewpoint is the study performed, potential impact of findings [2]. Briefly justify the choice of the comparator.

	1	2	3	4	5	
subitem not at all important	○	○	○	○	○	essential

Does your paper address subitem 2a-ii? *

Copy and paste relevant sections from the manuscript (include quotes in quotation marks "like this" to indicate direct quotes from your manuscript), or elaborate on this item by providing additional information not in the ms, or briefly explain why the item is not applicable/relevant for your study

"Virtual reality (VR) technology offers a controlled and flexible way to expose patients to feared stimuli, potentially overcoming some challenges of traditional exposure therapy [29,30]. Meta-analyses suggest that virtual reality exposure (VRE)-based treatments are as effective as active control conditions for anxiety disorders [31–33]. However, VRE has primarily been studied in individual therapy rather than group settings and has not been extensively evaluated in naturalistic clinical settings.
In clinical practice, therapy for anxiety disorders is often conducted in groups, as group therapy has shown efficacy comparable to individual therapy [15,34,35] while being considered more cost-effective. In Danish mental health services, SAD and agoraphobia without panic disorder are treated in standardized "mixed anxiety" CBT groups. The aim of the SoREAL trial was to evaluate the efficacy of VRE-based therapy for SAD and agoraphobia in a naturalistic clinical setting. As such, this study was embedded in the Danish mental health services, where VRE-based CBT (VR-CBT) was implemented in a mixed anxiety group format.
We hypothesized that VR-CBT would result in greater reductions in phobic anxiety compared to traditional CBT, as we expected the VRE format to allow for more tailored and higher dosages of exposure than in-vivo exposure therapy."

2b) In INTRODUCTION: Specific objectives or hypotheses

Does your paper address CONSORT subitem 2b? *

Copy and paste relevant sections from the manuscript (include quotes in quotation marks "like this" to indicate direct quotes from your manuscript), or elaborate on this item by providing additional information not in the ms, or briefly explain why the item is not applicable/relevant for your study

"We hypothesized that VR-CBT would result in greater reductions in phobic anxiety compared to traditional CBT, as we expected the VRE format to allow for more tailored and higher dosages of exposure than in-vivo exposure therapy."

METHODS

3a) Description of trial design (such as parallel, factorial) including allocation ratio

Does your paper address CONSORT subitem 3a? *

Copy and paste relevant sections from the manuscript (include quotes in quotation marks "like this" to indicate direct quotes from your manuscript), or elaborate on this item by providing additional information not in the ms, or briefly explain why the item is not applicable/relevant for your study

"We conducted a 1:1 randomized, assessor-blinded, parallel-group superiority trial across five outpatient psychotherapeutic clinics offering standardized anxiety group therapy within the Danish mental health services."

3b) Important changes to methods after trial commencement (such as eligibility criteria), with reasons

Does your paper address CONSORT subitem 3b? *

Copy and paste relevant sections from the manuscript (include quotes in quotation marks "like this" to indicate direct quotes from your manuscript), or elaborate on this item by providing additional information not in the ms, or briefly explain why the item is not applicable/relevant for your study

"The SoREAL trial was initially designed to investigate the efficacy of group VR-CBT for SAD (ICD code F40.1), and this design was registered at ClinicalTrials.gov as NCT03845101 before inclusion began. However, to address severe recruitment problems, we implemented several changes to the trial's design. We revised the inclusion criteria, removing the Liebowitz Social Anxiety Scale (LSAS) score requirement of >65 and adding agoraphobia (ICD code F40.0) as a possible primary diagnosis. Therapy manuals and VR software were modified with agoraphobia-specific modules and VR environments. The primary outcome measure was changed to percentage of maximum points (POMP)-transformed total scores of the LSAS for patients with SAD and the Mobility Inventory for Agoraphobia (MIA) for patients with agoraphobia. POMP transforming two different measures of anxiety allowed us to include patients with different primary diagnoses in the same analysis [37,38]. Finally, we expanded from two to five clinical sites and adjusted randomization to allocate therapy groups (rather than individual participants), stratified by site, to minimize wait times and account for the geographical distance between sites. The randomization module was updated in March 2020 after 40 participants had been individually randomized. The final trial design is also described in the published trial protocol [36]."

3b-i) Bug fixes, Downtimes, Content Changes

Bug fixes, Downtimes, Content Changes: ehealth systems are often dynamic systems. A description of changes to methods therefore also includes important changes made on the intervention or comparator during the trial (e.g., major bug fixes or changes in the functionality or content) (5-iii) and other "unexpected events" that may have influenced study design such as staff changes, system failures/downtimes, etc. [2].

	1	2	3	4	5	
subitem not at all important	○	○	○	◉	○	essential

Clear selection

Does your paper address subitem 3b-i?

Copy and paste relevant sections from the manuscript (include quotes in quotation marks "like this" to indicate direct quotes from your manuscript), or elaborate on this item by providing additional information not in the ms, or briefly explain why the item is not applicable/relevant for your study

"Therapy manuals and VR software were modified with agoraphobia-specific modules and VR environments."

4a) Eligibility criteria for participants

Does your paper address CONSORT subitem 4a? *

Copy and paste relevant sections from the manuscript (include quotes in quotation marks "like this" to indicate direct quotes from your manuscript), or elaborate on this item by providing additional information not in the ms, or briefly explain why the item is not applicable/relevant for your study

"Eligible participants had a primary diagnosis of SAD (ICD code F40.1) or agoraphobia (ICD code F40.0), were aged 18–75 years, had adequate proficiency in Danish, and were willing and able to provide informed consent. Patients were excluded if they had a drug or alcohol dependence."

4a-i) Computer / Internet literacy

Computer / Internet literacy is often an implicit "de facto" eligibility criterion - this should be explicitly clarified.

	1	2	3	4	5	
subitem not at all important	○	◉	○	○	○	essential

Clear selection

Does your paper address subitem 4a-i?

Copy and paste relevant sections from the manuscript (include quotes in quotation marks "like this" to indicate direct quotes from your manuscript), or elaborate on this item by providing additional information not in the ms, or briefly explain why the item is not applicable/relevant for your study

Not included since we wanted to test the intervention on a naturalistic population and as such designed the software to be as intuitive and easy to use as possible.

4a-ii) Open vs. closed, web-based vs. face-to-face assessments:

Open vs. closed, web-based vs. face-to-face assessments: Mention how participants were recruited (online vs. offline), e.g., from an open access website or from a clinic, and clarify if this was a purely web-based trial, or there were face-to-face components (as part of the intervention or for assessment), i.e., to what degree got the study team to know the participant. In online-only trials, clarify if participants were quasi-anonymous and whether having multiple identities was possible or whether technical or logistical measures (e.g., cookies, email confirmation, phone calls) were used to detect/prevent these.

	1	2	3	4	5	
subitem not at all important	○	○	○	○	◉	essential

Clear selection

Does your paper address subitem 4a-ii? *

Copy and paste relevant sections from the manuscript (include quotes in quotation marks "like this" to indicate direct quotes from your manuscript), or elaborate on this item by providing additional information not in the ms, or briefly explain why the item is not applicable/relevant for your study

"Patients were assessed before treatment, approximately two weeks after treatment, and nine months after treatment by trained assessors. Assessments took place at the clinics where patients received treatment or via telephone if necessary due to COVID-19 restrictions. Eligibility was confirmed by assessors during the baseline interview. Self-reported questionnaires were answered by hand (on printed copies) or online using a secure link."

4a-iii) Information giving during recruitment

Information given during recruitment. Specify how participants were briefed for recruitment and in the informed consent procedures (e.g., publish the informed consent documentation as appendix, see also item X26), as this information may have an effect on user self-selection, user expectation and may also bias results.

	1	2	3	4	5	
subitem not at all important	○	○	○	●	○	essential

Clear selection

Does your paper address subitem 4a-iii?

Copy and paste relevant sections from the manuscript (include quotes in quotation marks "like this" to indicate direct quotes from your manuscript), or elaborate on this item by providing additional information not in the ms, or briefly explain why the item is not applicable/relevant for your study

"and were willing and able to provide informed consent (See supplemental file 1). " We will publish participant information and consent form along with the manuscript.

4b) Settings and locations where the data were collected

Does your paper address CONSORT subitem 4b? *

Copy and paste relevant sections from the manuscript (include quotes in quotation marks "like this" to indicate direct quotes from your manuscript), or elaborate on this item by providing additional information not in the ms, or briefly explain why the item is not applicable/relevant for your study

"across five outpatient psychotherapeutic clinics offering standardized anxiety group therapy within the Danish mental health services."

"Assessments took place at the clinics where patients received treatment or via telephone if necessary due to COVID-19 restrictions."

4b-i) Report if outcomes were (self-)assessed through online questionnaires

Clearly report if outcomes were (self-)assessed through online questionnaires (as common in web-based trials) or otherwise.

	1	2	3	4	5	
subitem not at all important	○	○	○	○	◉	essential

Clear selection

Does your paper address subitem 4b-i? *

Copy and paste relevant sections from the manuscript (include quotes in quotation marks "like this" to indicate direct quotes from your manuscript), or elaborate on this item by providing additional information not in the ms, or briefly explain why the item is not applicable/relevant for your study

When outcomes can be both self-assesed or clinician-assessed, we state this: "The primary outcome was a reduction in phobic anxiety, measured with POMP-transformed total scores of the LSAS [46] (clinician-rated) "

4b-ii) Report how institutional affiliations are displayed

Report how institutional affiliations are displayed to potential participants [on ehealth media], as affiliations with prestigious hospitals or universities may affect volunteer rates, use, and reactions with regards to an intervention.(Not a required item – describe only if this may bias results)

	1	2	3	4	5	
subitem not at all important	○	◉	○	○	○	essential

Clear selection

Does your paper address subitem 4b-ii?

Copy and paste relevant sections from the manuscript (include quotes in quotation marks "like this" to indicate direct quotes from your manuscript), or elaborate on this item by providing additional information not in the ms, or briefly explain why the item is not applicable/relevant for your study

We clearly indicate that we are part of the Danish Mental Health Services

5) The interventions for each group with sufficient details to allow replication, including how and when they were actually administered

5-i) Mention names, credential, affiliations of the developers, sponsors, and owners

Mention names, credential, affiliations of the developers, sponsors, and owners [6] (if authors/evaluators are owners or developer of the software, this needs to be declared in a "Conflict of interest" section or mentioned elsewhere in the manuscript).

	1	2	3	4	5	
subitem not at all important	○	○	○	○	◉	essential

Clear selection

Does your paper address subitem 5-i?

Copy and paste relevant sections from the manuscript (include quotes in quotation marks "like this" to indicate direct quotes from your manuscript), or elaborate on this item by providing additional information not in the ms, or briefly explain why the item is not applicable/relevant for your study

"The exposure platform was developed collaboratively over 16 months with CBT experts, lived experience experts, and Danish VR developers “Khora”, who also own the platform"

"The Novo Nordisk Foundation awarded the SoREAL trial 5,000,000 DKK [NNF17OC0027780]. Additionally, the trial received a grant of 3,517,500 DKK from TrygFonden [ID: 146169]. The project is fully independent of both the Novo Nordisk Foundation and TrygFonden. Neither funding body influenced the study's design, data collection, analysis, interpretation, or manuscript preparation."

5-ii) Describe the history/development process

Describe the history/development process of the application and previous formative evaluations (e.g., focus groups, usability testing), as these will have an impact on adoption/use rates and help with interpreting results.

	1	2	3	4	5	
subitem not at all important	○	○	○	○	◉	essential

Clear selection

Does your paper address subitem 5-ii?

Copy and paste relevant sections from the manuscript (include quotes in quotation marks "like this" to indicate direct quotes from your manuscript), or elaborate on this item by providing additional information not in the ms, or briefly explain why the item is not applicable/relevant for your study

"The exposure platform was developed collaboratively over 16 months with CBT experts, lived experience experts, and Danish VR developers “Khora”, who also own the platform. Lived experience experts provided input on anxiety levels and scenario relevance, which guided adjustments. Details about the VR environments are available in supplemental file 2."

5-iii) Revisions and updating

Revisions and updating. Clearly mention the date and/or version number of the application/intervention (and comparator, if applicable) evaluated, or describe whether the intervention underwent major changes during the evaluation process, or whether the development and/or content was “frozen” during the trial. Describe dynamic components such as news feeds or changing content which may have an impact on the replicability of the intervention (for unexpected events see item 3b).

	1	2	3	4	5	
subitem not at all important	○	○	●	○	○	essential

Clear selection

Does your paper address subitem 5-iii?

Copy and paste relevant sections from the manuscript (include quotes in quotation marks "like this" to indicate direct quotes from your manuscript), or elaborate on this item by providing additional information not in the ms, or briefly explain why the item is not applicable/relevant for your study

"Therapy manuals and VR software were modified with agoraphobia-specific modules and VR environments."

5-iv) Quality assurance methods

Provide information on quality assurance methods to ensure accuracy and quality of information provided [1], if applicable.

	1	2	3	4	5	
subitem not at all important	○	○	○	●	○	essential

Clear selection

Does your paper address subitem 5-iv?

Copy and paste relevant sections from the manuscript (include quotes in quotation marks "like this" to indicate direct quotes from your manuscript), or elaborate on this item by providing additional information not in the ms, or briefly explain why the item is not applicable/relevant for your study

"Treatment fidelity was evaluated using a therapist self-report questionnaire administered five times throughout therapy, consisting of six Likert-style questions (ranging from 1 to 5) and questions about time spent on exposure therapy."

5-v) Ensure replicability by publishing the source code, and/or providing screenshots/screen-capture video, and/or providing flowcharts of the algorithms used

Ensure replicability by publishing the source code, and/or providing screenshots/screen-capture video, and/or providing flowcharts of the algorithms used. Replicability (i.e., other researchers should in principle be able to replicate the study) is a hallmark of scientific reporting.

	1	2	3	4	5	
subitem not at all important	○	○	○	◉	○	essential

Clear selection

Does your paper address subitem 5-v?

Copy and paste relevant sections from the manuscript (include quotes in quotation marks "like this" to indicate direct quotes from your manuscript), or elaborate on this item by providing additional information not in the ms, or briefly explain why the item is not applicable/relevant for your study

"Details about the VR environments are available in supplemental file 2." We cannot publish source code, since we do not own it. However, at its core, the program is a 360 video player. The core of the intervention is the videos which are linked to in the supplemental file and can be recreated.

5-vi) Digital preservation

Digital preservation: Provide the URL of the application, but as the intervention is likely to change or disappear over the course of the years; also make sure the intervention is archived (Internet Archive, webcitation.org, and/or publishing the source code or screenshots/videos alongside the article). As pages behind login screens cannot be archived, consider creating demo pages which are accessible without login.

	1	2	3	4	5	
subitem not at all important	○	○	○	◉	○	essential

Clear selection

Does your paper address subitem 5-vi?

Copy and paste relevant sections from the manuscript (include quotes in quotation marks "like this" to indicate direct quotes from your manuscript), or elaborate on this item by providing additional information not in the ms, or briefly explain why the item is not applicable/relevant for your study

There is no URL for the application. However the supplemental file has links to youtube videos that are unlikely to be taken down.

5-vii) Access

Access: Describe how participants accessed the application, in what setting/context, if they had to pay (or were paid) or not, whether they had to be a member of specific group. If known, describe how participants obtained "access to the platform and Internet" [1]. To ensure access for editors/reviewers/readers, consider to provide a "backdoor" login account or demo mode for reviewers/readers to explore the application (also important for archiving purposes, see vi).

	1	2	3	4	5	
subitem not at all important	○	○	◉	○	○	essential

Clear selection

Does your paper address subitem 5-vii? *

Copy and paste relevant sections from the manuscript (include quotes in quotation marks "like this" to indicate direct quotes from your manuscript), or elaborate on this item by providing additional information not in the ms, or briefly explain why the item is not applicable/relevant for your study

"Each patient received an Oculus Go head-mounted display and high-quality sound-blocking headphones for use throughout treatment. The VR environments were high-resolution, 360° stereoscopic films depicting salient, fear-inducing situations for SAD and agoraphobia. Patients chose from 13 scenarios with four to six scenes of increasing difficulty. Scenes transitioned to a looped, action-free sequence in the same environment to allow habituation as needed. Patients were introduced to the VR equipment and guided to select scenarios relevant to their anxieties. Exposure exercises were completed individually in the therapy room, with sessions typically including one or two breaks for group discussions. "

Treatment in the Danish Mental Healthcare sector is free of charge.

5-viii) Mode of delivery, features/functionalities/components of the intervention and comparator, and the theoretical framework

Describe mode of delivery, features/functionalities/components of the intervention and comparator, and the theoretical framework [6] used to design them (instructional strategy [1], behaviour change techniques, persuasive features, etc., see e.g., [7, 8] for terminology). This includes an in-depth description of the content (including where it is coming from and who developed it) [1]," whether [and how] it is tailored to individual circumstances and allows users to track their progress and receive feedback" [6]. This also includes a description of communication delivery channels and – if computer-mediated communication is a component – whether communication was synchronous or asynchronous [6]. It also includes information on presentation strategies [1], including page design principles, average amount of text on pages, presence of hyperlinks to other resources, etc. [1].

	1	2	3	4	5	
subitem not at all important	○	○	○	◉	○	essential

Clear selection

Does your paper address subitem 5-viii? *

Copy and paste relevant sections from the manuscript (include quotes in quotation marks "like this" to indicate direct quotes from your manuscript), or elaborate on this item by providing additional information not in the ms, or briefly explain why the item is not applicable/relevant for your study

"Both interventions followed a standardized structure and included the same core elements: one hour of individual therapy in preparation for group sessions, 14 weekly two-hour group therapy sessions, and optional components consisting of next-of-kin involvement, pharmacological treatment planning, physical therapy, and coordination with social services. The group therapy in both interventions was manualized and based on established CBT approaches to treating anxiety [41–44]. The manual was flexible, allowing clinicians to change the order of sessions. However, the amount of time allocated for exposure in both interventions was fixed, encompassing all related processes such as setup, transport, and preparation. Therapy groups consisted of eight to nine patients and were led by two clinicians trained in CBT (e.g., psychologists, medical doctors, or physiotherapists trained in psychotherapy). Acute supplementary individual sessions were also available in both interventions. In seven of the 14 sessions, 45 minutes were allocated to either VR or in-vivo exposure exercises. Both interventions included homework consisting of in-vivo exposure tasks between sessions and one session of "out-of-clinic" in-vivo exposure (e.g., taking the bus or going shopping). The exposure exercises aimed to foster adaptive responses to anxiety-provoking situations, reinforce cognitive restructuring, train general cognitive strategies (e.g., identifying negative automatic thoughts), and build social skills. The cognitive therapy strategies used in non-exposure sessions included psychoeducation, cognitive restructuring, shifting self-focused attention, and relapse prevention. The interventions differed only in the delivery method of exposure therapy."

5-ix) Describe use parameters

Describe use parameters (e.g., intended “doses” and optimal timing for use). Clarify what instructions or recommendations were given to the user, e.g., regarding timing, frequency, heaviness of use, if any, or was the intervention used ad libitum.

	1	2	3	4	5	
subitem not at all important	○	○	○	●	○	essential

Clear selection

Does your paper address subitem 5-ix?

Copy and paste relevant sections from the manuscript (include quotes in quotation marks "like this" to indicate direct quotes from your manuscript), or elaborate on this item by providing additional information not in the ms, or briefly explain why the item is not applicable/relevant for your study

"In seven of the 14 sessions, 45 minutes were allocated to either VR or in-vivo exposure exercises."

5-x) Clarify the level of human involvement

Clarify the level of human involvement (care providers or health professionals, also technical assistance) in the e-intervention or as co-intervention (detail number and expertise of professionals involved, if any, as well as “type of assistance offered, the timing and frequency of the support, how it is initiated, and the medium by which the assistance is delivered”. It may be necessary to distinguish between the level of human involvement required for the trial, and the level of human involvement required for a routine application outside of a RCT setting (discuss under item 21 – generalizability).

	1	2	3	4	5	
subitem not at all important	○	○	○	◉	○	essential

Clear selection

Does your paper address subitem 5-x?

Copy and paste relevant sections from the manuscript (include quotes in quotation marks "like this" to indicate direct quotes from your manuscript), or elaborate on this item by providing additional information not in the ms, or briefly explain why the item is not applicable/relevant for your study

"Therapy groups consisted of eight to nine patients and were led by two clinicians trained in CBT (e.g., psychologists, medical doctors, or physiotherapists trained in psychotherapy). Acute supplementary individual sessions were also available in both interventions. "

5-xi) Report any prompts/reminders used

Report any prompts/reminders used: Clarify if there were prompts (letters, emails, phone calls, SMS) to use the application, what triggered them, frequency etc. It may be necessary to distinguish between the level of prompts/reminders required for the trial, and the level of prompts/reminders for a routine application outside of a RCT setting (discuss under item 21 – generalizability).

	1	2	3	4	5	
subitem not at all important	○	○	○	◉	○	essential

Clear selection

Does your paper address subitem 5-xi? *

Copy and paste relevant sections from the manuscript (include quotes in quotation marks "like this" to indicate direct quotes from your manuscript), or elaborate on this item by providing additional information not in the ms, or briefly explain why the item is not applicable/relevant for your study

Not relevant. Patients only used the software while in group therapy.

5-xii) Describe any co-interventions (incl. training/support)

Describe any co-interventions (incl. training/support): Clearly state any interventions that are provided in addition to the targeted eHealth intervention, as ehealth intervention may not be designed as stand-alone intervention. This includes training sessions and support [1]. It may be necessary to distinguish between the level of training required for the trial, and the level of training for a routine application outside of a RCT setting (discuss under item 21 – generalizability.

	1	2	3	4	5	
subitem not at all important	○	○	○	◉	○	essential

Clear selection

Does your paper address subitem 5-xii? *

Copy and paste relevant sections from the manuscript (include quotes in quotation marks "like this" to indicate direct quotes from your manuscript), or elaborate on this item by providing additional information not in the ms, or briefly explain why the item is not applicable/relevant for your study

"and optional components consisting of next-of-kin involvement, pharmacological treatment planning, physical therapy, and coordination with social services."

This is considered part of the intervention, but since they are optional, not all patients will recieve them, therefore we state them here under co-interventions.

6a) Completely defined pre-specified primary and secondary outcome measures, including how and when they were assessed

Does your paper address CONSORT subitem 6a? *

Copy and paste relevant sections from the manuscript (include quotes in quotation marks "like this" to indicate direct quotes from your manuscript), or elaborate on this item by providing additional information not in the ms, or briefly explain why the item is not applicable/relevant for your study

"Patients were assessed before treatment (baseline), approximately two weeks after treatment (post-treatment), and nine months after treatment start (follow-up) by trained assessors.

Primary diagnoses and comorbidities were assessed with the Mini-International Neuropsychiatric Interview version 7 [45]. The primary outcome was a reduction in phobic anxiety, measured with POMP-transformed total scores of the LSAS [46] (clinician-rated) for patients with SAD and the MIA [47] for patients with agoraphobia.

"Secondary outcomes included fear of negative evaluation, measured with the Brief Version of the Fear of Negative Evaluation Scale [48]; depression symptoms, measured with the six-item version of the Hamilton Depression Rating Scale [49]; work and social functioning, measured with the Work and Social Adjustment Scale [50]; treatment satisfaction, measured with the Client Satisfaction Questionnaire [51]; and quality of life, measured with the WHO-5 Well-Being Index [52]. SAD treatment response was defined as an LSAS score below 50 or a reduction of 15 points, and remission was defined as an LSAS score below 25. For agoraphobia, treatment response was defined as an MIA score below 2 or a reduction of 0.5 points, and remission was defined as an MIA score below 1.5, assessed post-treatment and at follow-up.

Exploratory outcomes included social functioning, measured with the Personal and Social Performance Scale [53]; substance and alcohol use over the last month, measured with Timeline Followback [54]; self-efficacy, measured with the General Self-Efficacy Scale [55]; working alliance, measured with the Working Alliance Inventory [56]; SAD symptom severity for patients with SAD as a primary diagnosis, measured with the LSAS (clinician-rated); and agoraphobia symptoms for patients with agoraphobia as a primary diagnosis, measured with the MIA.

Other measures included simulation sickness from VR, assessed using the Simulation Sickness Questionnaire [57] after each exposure session. Deterioration and adverse effects of psychotherapy on SAD symptoms were defined as an increase of 6.8 points or more on the LSAS total score, while for agoraphobia, deterioration was defined as an increase of 0.3 points or more on the MIA total score, assessed post-treatment and at follow-up. Treatment fidelity was evaluated using a therapist self-report questionnaire administered five times throughout therapy, consisting of six Likert-style questions (ranging from 1 to 5) and questions about time spent on exposure therapy."

6a-i) Online questionnaires: describe if they were validated for online use and apply CHERRIES items to describe how the questionnaires were designed/deployed

If outcomes were obtained through online questionnaires, describe if they were validated for online use and apply CHERRIES items to describe how the questionnaires were designed/deployed [9].

	1	2	3	4	5	
subitem not at all important	○	○	◉	○	○	essential

Clear selection

Does your paper address subitem 6a-i?

Copy and paste relevant sections from manuscript text

Participants had the option to complete self-report questionnaries on paper or via a link. All self-report questionnaires have been used extensively with electronic delivery mechanisms.

6a-ii) Describe whether and how “use” (including intensity of use/dosage) was defined/measured/monitored

Describe whether and how “use” (including intensity of use/dosage) was defined/measured/monitored (logins, logfile analysis, etc.). Use/adoption metrics are important process outcomes that should be reported in any ehealth trial.

	1	2	3	4	5	
subitem not at all important	○	○	○	◉	○	essential

Clear selection

Does your paper address subitem 6a-ii?

Copy and paste relevant sections from manuscript text

"Treatment fidelity was evaluated using a therapist self-report questionnaire administered five times throughout therapy, consisting of six Likert-style questions (ranging from 1 to 5) and questions about time spent on exposure therapy."

6a-iii) Describe whether, how, and when qualitative feedback from participants was obtained

Describe whether, how, and when qualitative feedback from participants was obtained (e.g., through emails, feedback forms, interviews, focus groups).

	1	2	3	4	5	
subitem not at all important	○	○	◉	○	○	essential

Clear selection

Does your paper address subitem 6a-iii?

Copy and paste relevant sections from manuscript text

We conducted a qualitative study but results are published elsewhere.

6b) Any changes to trial outcomes after the trial commenced, with reasons

Does your paper address CONSORT subitem 6b? *

Copy and paste relevant sections from the manuscript (include quotes in quotation marks "like this" to indicate direct quotes from your manuscript), or elaborate on this item by providing additional information not in the ms, or briefly explain why the item is not applicable/relevant for your study

"Design changes after trial registration
The SoREAL trial was initially designed to investigate the efficacy of group VR-CBT for SAD (ICD code F40.1), and this design was registered at ClinicalTrials.gov as NCT03845101 before inclusion began. However, to address severe recruitment problems, we implemented several changes to the trial's design. We revised the inclusion criteria, removing the Liebowitz Social Anxiety Scale (LSAS) score requirement of >65 and adding agoraphobia (ICD code F40.0) as a possible primary diagnosis. Therapy manuals and VR software were modified with agoraphobia-specific modules and VR environments. The primary outcome measure was changed to percentage of maximum points (POMP)-transformed total scores of the LSAS for patients with SAD and the Mobility Inventory for Agoraphobia (MIA) for patients with agoraphobia. POMP transforming two different measures of anxiety allowed us to include patients with different primary diagnoses in the same analysis [37,38]. Finally, we expanded from two to five clinical sites and adjusted randomization to allocate therapy groups (rather than individual participants), stratified by site, to minimize wait times and account for the geographical distance between sites. The randomization module was updated in March 2020 after 40 participants had been individually randomized. The final trial design is also described in the published trial protocol [36]."

7a) How sample size was determined

NPT: When applicable, details of whether and how the clustering by care provides or centers was addressed

7a-i) Describe whether and how expected attrition was taken into account when calculating the sample size

Describe whether and how expected attrition was taken into account when calculating the sample size.

	1	2	3	4	5	
subitem not at all important	○	○	○	○	●	essential

Clear selection

Does your paper address subitem 7a-i?

Copy and paste relevant sections from manuscript title (include quotes in quotation marks "like this" to indicate direct quotes from your manuscript), or elaborate on this item by providing additional information not in the ms, or briefly explain why the item is not applicable/relevant for your study

To keep the paper succinct, we state the following with a reference to our protocol paper which has more detailed descriptions of our power calculations

"We estimated that 302 participants were needed to detect the minimal clinically relevant differences in phobic anxiety severity reductions between groups [36]."

7b) When applicable, explanation of any interim analyses and stopping guidelines

Does your paper address CONSORT subitem 7b? *

Copy and paste relevant sections from the manuscript (include quotes in quotation marks "like this" to indicate direct quotes from your manuscript), or elaborate on this item by providing additional information not in the ms, or briefly explain why the item is not applicable/relevant for your study

We did not conduct any interim analyses and did not have pre-formulated stopping guidelines.

8a) Method used to generate the random allocation sequence

NPT: When applicable, how care providers were allocated to each trial group

Does your paper address CONSORT subitem 8a? *

Copy and paste relevant sections from the manuscript (include quotes in quotation marks "like this" to indicate direct quotes from your manuscript), or elaborate on this item by providing additional information not in the ms, or briefly explain why the item is not applicable/relevant for your study

"Allocation tables were generated by a statistician using an online tool [40]"

8b) Type of randomisation; details of any restriction (such as blocking and block size)

Does your paper address CONSORT subitem 8b? *

Copy and paste relevant sections from the manuscript (include quotes in quotation marks "like this" to indicate direct quotes from your manuscript), or elaborate on this item by providing additional information not in the ms, or briefly explain why the item is not applicable/relevant for your study

"Therapy groups were randomly assigned (1:1)" "varying block sizes of two and four and stratification by treatment site".

9) Mechanism used to implement the random allocation sequence (such as sequentially numbered containers), describing any steps taken to conceal the sequence until interventions were assigned

Does your paper address CONSORT subitem 9? *

Copy and paste relevant sections from the manuscript (include quotes in quotation marks "like this" to indicate direct quotes from your manuscript), or elaborate on this item by providing additional information not in the ms, or briefly explain why the item is not applicable/relevant for your study

" Randomization was performed using REDCap [39] by a trial coordinator uninvolved in data collection, who informed the relevant clinicians via email or telephone. "

10) Who generated the random allocation sequence, who enrolled participants, and who assigned participants to interventions

Does your paper address CONSORT subitem 10? *

Copy and paste relevant sections from the manuscript (include quotes in quotation marks "like this" to indicate direct quotes from your manuscript), or elaborate on this item by providing additional information not in the ms, or briefly explain why the item is not applicable/relevant for your study

"Randomization was performed using REDCap [39] by a trial coordinator uninvolved in data collection, who informed the relevant clinicians via email or telephone. Allocation tables were generated by a statistician"
"Trial assessors were blinded to group allocation, and if unblinded, another assessor conducted the assessment."

11a) If done, who was blinded after assignment to interventions (for example, participants, care providers, those assessing outcomes) and how

NPT: Whether or not administering co-interventions were blinded to group assignment

11a-i) Specify who was blinded, and who wasn't

Specify who was blinded, and who wasn't. Usually, in web-based trials it is not possible to blind the participants [1, 3] (this should be clearly acknowledged), but it may be possible to blind outcome assessors, those doing data analysis or those administering co-interventions (if any).

	1	2	3	4	5	
subitem not at all important	○	○	○	●	○	essential

Clear selection

Does your paper address subitem 11a-i? *

Copy and paste relevant sections from the manuscript (include quotes in quotation marks "like this" to indicate direct quotes from your manuscript), or elaborate on this item by providing additional information not in the ms, or briefly explain why the item is not applicable/relevant for your study

"Trial assessors were blinded to group allocation, and if unblinded, another assessor conducted the assessment."

11a-ii) Discuss e.g., whether participants knew which intervention was the "intervention of interest" and which one was the "comparator"

Informed consent procedures (4a-ii) can create biases and certain expectations - discuss e.g., whether participants knew which intervention was the "intervention of interest" and which one was the "comparator".

	1	2	3	4	5	
subitem not at all important	○	○	◉	○	○	essential

Clear selection

Does your paper address subitem 11a-ii?

Copy and paste relevant sections from the manuscript (include quotes in quotation marks "like this" to indicate direct quotes from your manuscript), or elaborate on this item by providing additional information not in the ms, or briefly explain why the item is not applicable/relevant for your study

It was not feasible to blind participants to group assignment given that one group recieved VR treatment and the other did not

11b) If relevant, description of the similarity of interventions

(this item is usually not relevant for ehealth trials as it refers to similarity of a placebo or sham intervention to a active medication/intervention)

Does your paper address CONSORT subitem 11b? *

Copy and paste relevant sections from the manuscript (include quotes in quotation marks "like this" to indicate direct quotes from your manuscript), or elaborate on this item by providing additional information not in the ms, or briefly explain why the item is not applicable/relevant for your study

"The interventions differed only in the delivery method of exposure therapy."

12a) Statistical methods used to compare groups for primary and secondary outcomes

NPT: When applicable, details of whether and how the clustering by care providers or centers was addressed

Does your paper address CONSORT subitem 12a? *

Copy and paste relevant sections from the manuscript (include quotes in quotation marks "like this" to indicate direct quotes from your manuscript), or elaborate on this item by providing additional information not in the ms, or briefly explain why the item is not applicable/relevant for your study

"Statistical analysis
We aimed to enroll 302 patients based on the following parameters: an alpha of 0.05, 80% power, and a standard deviation of 21 to detect a difference of 6.8 on the LSAS, corresponding to a Cohen’s d of 0.33. After expanding inclusion to patients with agoraphobia, we retained the target N of 302, even though the lower variability of the MIA would have reduced the required sample size. All analyses were conducted using an intention-to-treat approach, with tests performed at a two-tailed 5% level of significance. Missing data were handled using multiple imputations (m=100), with predictors in the imputation model including baseline and post-treatment scores of primary, secondary, and exploratory outcomes; primary diagnosis (SAD or agoraphobia); degree of comorbidity; and session attendance. Group differences were examined using analysis of covariance (ANCOVA), with primary diagnosis, degree of comorbidity, and baseline severity as covariates. For POMP-transformed outcomes and analyses of specific diagnostic groups, primary diagnosis was not included as a covariate. Assumptions for ANCOVA were checked as follows: normality of the dependent variable via visual inspection of Q–Q plots, homogeneity of variance using Levene’s test, and homogeneity of regression slopes with univariate analysis of variance. Baseline severity was excluded as a covariate for post-treatment analyses of the Fear of Negative Evaluation and Work and Social Adjustment Scales, and post-treatment severity was excluded as a covariate for follow-up analysis of the Hamilton Depression Rating Scale due to non-homogeneity of regression slopes. For dichotomous outcomes, multiple logistic regression analyses were performed using primary diagnosis, comorbidity, and baseline severity as predictors. Model fit was assessed using Hosmer-Lemeshow’s test, and multicollinearity was evaluated using tolerance values and variance inflation factors. Due to the small number of drug use incidents (<5 in both groups), drug use was not analyzed."

12a-i) Imputation techniques to deal with attrition / missing values

Imputation techniques to deal with attrition / missing values: Not all participants will use the intervention/comparator as intended and attrition is typically high in ehealth trials. Specify how participants who did not use the application or dropped out from the trial were treated in the statistical analysis (a complete case analysis is strongly discouraged, and simple imputation techniques such as LOCF may also be problematic [4]).

	1	2	3	4	5	
subitem not at all important	○	○	○	◉	○	essential

Clear selection

Does your paper address subitem 12a-i? *

Copy and paste relevant sections from the manuscript (include quotes in quotation marks "like this" to indicate direct quotes from your manuscript), or elaborate on this item by providing additional information not in the ms, or briefly explain why the item is not applicable/relevant for your study

"Missing data were handled using multiple imputations (m=100), with predictors in the imputation model including baseline and post-treatment scores of primary, secondary, and exploratory outcomes; primary diagnosis (SAD or agoraphobia); degree of comorbidity; and session attendance."

12b) Methods for additional analyses, such as subgroup analyses and adjusted analyses

Does your paper address CONSORT subitem 12b? *

Copy and paste relevant sections from the manuscript (include quotes in quotation marks "like this" to indicate direct quotes from your manuscript), or elaborate on this item by providing additional information not in the ms, or briefly explain why the item is not applicable/relevant for your study

We do not report any subsgroup analyses or adjusted analyses in this paper

X26) REB/IRB Approval and Ethical Considerations [recommended as subheading under "Methods"] (not a CONSORT item)

X26-i) Comment on ethics committee approval

	1	2	3	4	5	
subitem not at all important	○	○	○	◉	○	essential

Clear selection

Does your paper address subitem X26-i?

Copy and paste relevant sections from the manuscript (include quotes in quotation marks "like this" to indicate direct quotes from your manuscript), or elaborate on this item by providing additional information not in the ms, or briefly explain why the item is not applicable/relevant for your study

"Statement of Ethics
The SoREAL trial received approval from the Regional Ethics Committee of Zealand (H-6-2013-015) and the Danish Data Protection Agency (RHP2014-009-02670). All participants were informed about the study procedures and provided written informed consent prior to participation. The individuals appearing in the supplemental material are professional actors who were hired for this study. Written consent has been obtained from all actors for the use of their images in this publication."

x26-ii) Outline informed consent procedures

Outline informed consent procedures e.g., if consent was obtained offline or online (how? Checkbox, etc.?), and what information was provided (see 4a-ii). See [6] for some items to be included in informed consent documents.

	1	2	3	4	5	
subitem not at all important	○	○	◉	○	○	essential

Clear selection

Does your paper address subitem X26-ii?

Copy and paste relevant sections from the manuscript (include quotes in quotation marks "like this" to indicate direct quotes from your manuscript), or elaborate on this item by providing additional information not in the ms, or briefly explain why the item is not applicable/relevant for your study

All participants were informed about the study procedures and provided written informed consent prior to participation.

X26-iii) Safety and security procedures

Safety and security procedures, incl. privacy considerations, and any steps taken to reduce the likelihood or detection of harm (e.g., education and training, availability of a hotline)

	1	2	3	4	5	
subitem not at all important	○	○	○	◉	○	essential

Clear selection

Does your paper address subitem X26-iii?

Copy and paste relevant sections from the manuscript (include quotes in quotation marks "like this" to indicate direct quotes from your manuscript), or elaborate on this item by providing additional information not in the ms, or briefly explain why the item is not applicable/relevant for your study

We measured simulation sickness after every session where VR was used

RESULTS

13a) For each group, the numbers of participants who were randomly assigned, received intended treatment, and were analysed for the primary outcome

NPT: The number of care providers or centers performing the intervention in each group and the number of patients treated by each care provider in each center

Does your paper address CONSORT subitem 13a? *

Copy and paste relevant sections from the manuscript (include quotes in quotation marks "like this" to indicate direct quotes from your manuscript), or elaborate on this item by providing additional information not in the ms, or briefly explain why the item is not applicable/relevant for your study

" Figure 1 illustrates the recruitment and assessment flow of the trial."

13b) For each group, losses and exclusions after randomisation, together with reasons

Does your paper address CONSORT subitem 13b? (NOTE: Preferably, this is shown in a CONSORT flow diagram) *

Copy and paste relevant sections from the manuscript (include quotes in quotation marks "like this" to indicate direct quotes from your manuscript), or elaborate on this item by providing additional information not in the ms, or briefly explain why the item is not applicable/relevant for your study

" Figure 1 illustrates the recruitment and assessment flow of the trial."

13b-i) Attrition diagram

Strongly recommended: An attrition diagram (e.g., proportion of participants still logging in or using the intervention/comparator in each group plotted over time, similar to a survival curve) or other figures or tables demonstrating usage/dose/engagement.

	1	2	3	4	5	
subitem not at all important	○	○	◉	○	○	essential

Clear selection

Does your paper address subitem 13b-i?

Copy and paste relevant sections from the manuscript or cite the figure number if applicable (include quotes in quotation marks "like this" to indicate direct quotes from your manuscript), or elaborate on this item by providing additional information not in the ms, or briefly explain why the item is not applicable/relevant for your study

Not relevant for our intervention

14a) Dates defining the periods of recruitment and follow-up

Does your paper address CONSORT subitem 14a? *

Copy and paste relevant sections from the manuscript (include quotes in quotation marks "like this" to indicate direct quotes from your manuscript), or elaborate on this item by providing additional information not in the ms, or briefly explain why the item is not applicable/relevant for your study

"Patients were assessed before treatment (baseline), approximately two weeks after treatment (post-treatment), and nine months after treatment start (follow-up) by trained assessors. "

14a-i) Indicate if critical “secular events” fell into the study period

Indicate if critical “secular events” fell into the study period, e.g., significant changes in Internet resources available or “changes in computer hardware or Internet delivery resources”

	1	2	3	4	5	
subitem not at all important	○	○	◉	○	○	essential

Clear selection

Does your paper address subitem 14a-i?

Copy and paste relevant sections from the manuscript (include quotes in quotation marks "like this" to indicate direct quotes from your manuscript), or elaborate on this item by providing additional information not in the ms, or briefly explain why the item is not applicable/relevant for your study

"Assessments took place at the clinics where patients received treatment or via telephone if necessary due to COVID-19 restrictions."

14b) Why the trial ended or was stopped (early)

Does your paper address CONSORT subitem 14b? *

Copy and paste relevant sections from the manuscript (include quotes in quotation marks "like this" to indicate direct quotes from your manuscript), or elaborate on this item by providing additional information not in the ms, or briefly explain why the item is not applicable/relevant for your study

"We estimated that 302 participants were needed to detect the minimal clinically relevant differences in phobic anxiety severity reductions between groups [36]. Participant recruitment began in February 2019 and ended prematurely in December 2023 due to recruitment and feasibility issues, resulting in a sample size of 177. These issues are elaborated in the discussion section."

"Failure to reach N
Being fully embedded in the Danish Mental Health Services, the SoREAL trial could only recruit patients referred by their GPs to the standardized anxiety treatment package. Based on clinical intake and experience from previous trials at the two initial sites, we expected to reach N in 36 months. However, changes to GP guidelines for psychiatric referrals, designed to reduce the case load of psychotherapeutic outpatient hospitals, resulted in most SAD patients being referred to practicing psychologists instead. To offset the slower-than-anticipated recruitment rate, we expanded the study to include three additional clinic sites, which also entailed the design changes described elsewhere.

Shortly after including the new sites, Denmark entered lockdown due to the COVID-19 pandemic. This meant that psychotherapeutic treatment was conducted via telephone and/or individually. A notable drop in referrals to treatment sites also occurred, possibly because SAD and agoraphobia symptoms are less functionally impairing during a national lockdown.

After the lockdown ended, the clinical sites decided not to continue with the trial for several reasons. The primary concern raised by the clinics was the ethicality of continuing a treatment perceived as unsuitable for the population and group format. These concerns stemmed from observations that patients found VR distressing in a group setting and struggled to focus on VR scenarios due to the social dynamics within the therapy room. Clinicians also raised concerns about the lack of interactivity in the virtual environments, which made it difficult to expose patients to core SAD fears, such as conversation. These concerns are consistent with prior findings [57] and align with the notion that therapeutic context, patient characteristics, and VR characteristics can influence the experience of VR environments [58,59]. Group settings, in particular, may introduce stressors like performance anxiety, which could potentially diminish immersion and thus the therapeutic value of VR environments. Addressing these concerns may require the use of VR technology that accounts for group settings and the need for flexible social interactions. Examples include multi-user VR, in which all patients engage in the same shared virtual environments; augmented reality, where the therapy room remains visible but virtual objects are overlaid to simulate therapeutic scenarios; or large language model-based avatars to engage in conversation.

Beyond this, staff turnover limited clinicians’ ability to gain experience with VR-CBT. Overall, 39 therapists delivered VR-CBT, most of whom could only receive very limited training in conducting VRE due to time constraints. Frequent technical malfunctions and limited time for in-session troubleshooting also impacted feasibility. These factors likely contributed to the low mean exposure time per VR-CBT group (85 minutes).

Lastly, the extended inclusion period strained the resources of the clinics, who had agreed to participate for limited periods (e.g., two years) and had obligations to other clinical trials. The factors that led clinics to opt out of the trial are important to consider for future studies examining VR-based psychotherapy in naturalistic clinical settings. They also underscore the importance of pilot studies for both design and intervention feasibility [57], which could have identified challenges related to recruitment rates, the group format, technical issues,

[illegible]
and clinician training requirements before conducting the full-scale RCT."

15) A table showing baseline demographic and clinical characteristics for each group
NPT: When applicable, a description of care providers (case volume, qualification, expertise, etc.) and centers (volume) in each group

Does your paper address CONSORT subitem 15? *

Copy and paste relevant sections from the manuscript (include quotes in quotation marks "like this" to indicate direct quotes from your manuscript), or elaborate on this item by providing additional information not in the ms, or briefly explain why the item is not applicable/relevant for your study

"A total of 177 participants were analysed, with 81 randomized to VR-CBT and 96 to CBT. Sociodemographic and clinical characteristics were balanced between groups (Table 1). "

15-i) Report demographics associated with digital divide issues

In ehealth trials it is particularly important to report demographics associated with digital divide issues, such as age, education, gender, social-economic status, computer/Internet/ehealth literacy of the participants, if known.

	1	2	3	4	5	
subitem not at all important	○	○	○	○	○	essential

Does your paper address subitem 15-i? *

Copy and paste relevant sections from the manuscript (include quotes in quotation marks "like this" to indicate direct quotes from your manuscript), or elaborate on this item by providing additional information not in the ms, or briefly explain why the item is not applicable/relevant for your study

"A total of 177 participants were analysed, with 81 randomized to VR-CBT and 96 to CBT. Sociodemographic and clinical characteristics were balanced between groups (Table 1). "

16) For each group, number of participants (denominator) included in each analysis and whether the analysis was by original assigned groups

16-i) Report multiple "denominators" and provide definitions

Report multiple "denominators" and provide definitions: Report N's (and effect sizes) "across a range of study participation [and use] thresholds" [1], e.g., N exposed, N consented, N used more than x times, N used more than y weeks, N participants "used" the intervention/comparator at specific pre-defined time points of interest (in absolute and relative numbers per group). Always clearly define "use" of the intervention.

	1	2	3	4	5	
subitem not at all important	○	○	○	●	○	essential

Clear selection

Does your paper address subitem 16-i? *

Copy and paste relevant sections from the manuscript (include quotes in quotation marks "like this" to indicate direct quotes from your manuscript), or elaborate on this item by providing additional information not in the ms, or briefly explain why the item is not applicable/relevant for your study

Not relevant for our study design which had a predetermined statistical analysis plan and a structured clinical intervention

16-ii) Primary analysis should be intent-to-treat

Primary analysis should be intent-to-treat, secondary analyses could include comparing only "users", with the appropriate caveats that this is no longer a randomized sample (see 18-i).

	1	2	3	4	5	
subitem not at all important	○	○	○	○	●	essential

Clear selection

Does your paper address subitem 16-ii?

Copy and paste relevant sections from the manuscript (include quotes in quotation marks "like this" to indicate direct quotes from your manuscript), or elaborate on this item by providing additional information not in the ms, or briefly explain why the item is not applicable/relevant for your study

Yes. We clearly indicate we conduct ITT analysis

17a) For each primary and secondary outcome, results for each group, and the estimated effect size and its precision (such as 95% confidence interval)

Does your paper address CONSORT subitem 17a? *

Copy and paste relevant sections from the manuscript (include quotes in quotation marks "like this" to indicate direct quotes from your manuscript), or elaborate on this item by providing additional information not in the ms, or briefly explain why the item is not applicable/relevant for your study

"Both groups showed reductions in phobic anxiety severity. At post-treatment, the VR-CBT group had an adjusted mean POMP score of 47.4 (SE=2.3), and the CBT group had an adjusted mean of 46.8 (SE=2.3), with no significant between-group differences (P=.61). No statistically significant differences were observed between groups at follow-up, with an adjusted mean of 43.6 (SE=1.8) for VR-CBT and 45.2 (SE=1.7) for CBT (P=.47) (Table 3)."

"Secondary Outcomes
Depressive symptoms decreased in both groups, with no statistically significant between-group differences (post-treatment adjusted means: VR-CBT=4.8, SE=0.4; CBT=5.1, SE=0.4; P=.66; follow-up adjusted means: VR-CBT=5.1, SE=0.6; CBT=5.3, SE=0.5; P=.77). Fear of negative evaluation also decreased similarly in both groups (post-treatment adjusted means: VR-CBT=3.7, SE=0.1; CBT=3.6, SE=0.1; P=.65; follow-up adjusted means: VR-CBT=3.7, SE=0.1; CBT=3.8, SE=0.1; P=.73) (Tables 3 and 4). Work and social adjustment scores improved slightly in both groups, with no significant between-group differences (post-treatment adjusted means: VR-CBT=3.7, SE=0.2; CBT=3.5, SE=0.2; P=.50; follow-up adjusted means: VR-CBT=3.4, SE=0.3; CBT=3.4, SE=0.2; P=.95). Quality of life also improved, but differences between groups were not statistically significant (post-treatment adjusted means: VR-CBT=41.5, SE=2.9; CBT=43.4, SE=2.8; P=.62; follow-up adjusted means: VR-CBT=42.8, SE=3.5; CBT=43.1, SE=2.9; P=.99) (Tables 3 and 4). At post-treatment, the odds of treatment response did not differ significantly between groups (P=.65), and no statistically significant differences were observed for the odds of remission at post-treatment (P=.49) or follow-up (P=.51) (Table 5)."

17a-i) Presentation of process outcomes such as metrics of use and intensity of use

In addition to primary/secondary (clinical) outcomes, the presentation of process outcomes such as metrics of use and intensity of use (dose, exposure) and their operational definitions is critical. This does not only refer to metrics of attrition (13-b) (often a binary variable), but also to more continuous exposure metrics such as “average session length”. These must be accompanied by a technical description how a metric like a “session” is defined (e.g., timeout after idle time) [1] (report under item 6a).

	1	2	3	4	5	
subitem not at all important	○	○	◉	○	○	essential

Clear selection

Does your paper address subitem 17a-i?

Copy and paste relevant sections from the manuscript (include quotes in quotation marks "like this" to indicate direct quotes from your manuscript), or elaborate on this item by providing additional information not in the ms, or briefly explain why the item is not applicable/relevant for your study

"The average total exposure time per therapy course was not statistically significantly different between groups (P=.09): 85 minutes for VR-CBT groups (SD=76.5) and 120.7 minutes for CBT groups (SD=76.5), which were overall less than a third of the 360 minutes specified in the treatment manuals. Therapist-reported fidelity scores did not differ significantly between groups (P=.24), with a mean of 4.0 (SD=0.3) for VR-CBT and 4.1 (SD=0.5) for CBT, suggesting that therapists adhered closely to the manualized treatment protocols. Among participants receiving VR-CBT, the mean simulation sickness score was high, with an average score of 36.2 (SD=27.9) across 51 participants. There were no reported serious adverse events or reactions."

17b) For binary outcomes, presentation of both absolute and relative effect sizes is recommended

Does your paper address CONSORT subitem 17b? *

Copy and paste relevant sections from the manuscript (include quotes in quotation marks "like this" to indicate direct quotes from your manuscript), or elaborate on this item by providing additional information not in the ms, or briefly explain why the item is not applicable/relevant for your study

Our statistical analysis plan specified that we would only conduct group comparisons and therefore we only report relative effect sizes

18) Results of any other analyses performed, including subgroup analyses and adjusted analyses, distinguishing pre-specified from exploratory

Does your paper address CONSORT subitem 18? *

Copy and paste relevant sections from the manuscript (include quotes in quotation marks "like this" to indicate direct quotes from your manuscript), or elaborate on this item by providing additional information not in the ms, or briefly explain why the item is not applicable/relevant for your study

We clearly describe exploratory outcomes and other measures

18-i) Subgroup analysis of comparing only users

A subgroup analysis of comparing only users is not uncommon in ehealth trials, but if done, it must be stressed that this is a self-selected sample and no longer an unbiased sample from a randomized trial (see 16-iii).

	1	2	3	4	5	
subitem not at all important	○	○	◉	○	○	essential

Clear selection

Does your paper address subitem 18-i?

Copy and paste relevant sections from the manuscript (include quotes in quotation marks "like this" to indicate direct quotes from your manuscript), or elaborate on this item by providing additional information not in the ms, or briefly explain why the item is not applicable/relevant for your study

No - it would have been relevant, but we did not specify this in the analysis plan of our trial protocol

19) All important harms or unintended effects in each group
(for specific guidance see CONSORT for harms)

Does your paper address CONSORT subitem 19? *

Copy and paste relevant sections from the manuscript (include quotes in quotation marks "like this" to indicate direct quotes from your manuscript), or elaborate on this item by providing additional information not in the ms, or briefly explain why the item is not applicable/relevant for your study

Yes, we state that no adverse events were reported and note simulation sickness scores.

19-i) Include privacy breaches, technical problems

Include privacy breaches, technical problems. This does not only include physical “harm” to participants, but also incidents such as perceived or real privacy breaches [1], technical problems, and other unexpected/unintended incidents. “Unintended effects” also includes unintended positive effects [2].

	1	2	3	4	5	
subitem not at all important	○	○	○	●	○	essential

Clear selection

Does your paper address subitem 19-i?

Copy and paste relevant sections from the manuscript (include quotes in quotation marks "like this" to indicate direct quotes from your manuscript), or elaborate on this item by providing additional information not in the ms, or briefly explain why the item is not applicable/relevant for your study

In the discussion section, we discuss technical issues encountered.

19-ii) Include qualitative feedback from participants or observations from staff/researchers

Include qualitative feedback from participants or observations from staff/researchers, if available, on strengths and shortcomings of the application, especially if they point to unintended/unexpected effects or uses. This includes (if available) reasons for why people did or did not use the application as intended by the developers.

	1	2	3	4	5	
subitem not at all important	○	○	○	◉	○	essential

Clear selection

Does your paper address subitem 19-ii?

Copy and paste relevant sections from the manuscript (include quotes in quotation marks "like this" to indicate direct quotes from your manuscript), or elaborate on this item by providing additional information not in the ms, or briefly explain why the item is not applicable/relevant for your study

We have published a qualitative analysis in a seperate paper

DISCUSSION

22) Interpretation consistent with results, balancing benefits and harms, and considering other relevant evidence

NPT: In addition, take into account the choice of the comparator, lack of or partial blinding, and unequal expertise of care providers or centers in each group

22-i) Restate study questions and summarize the answers suggested by the data, starting with primary outcomes and process outcomes (use)

Restate study questions and summarize the answers suggested by the data, starting with primary outcomes and process outcomes (use).

	1	2	3	4	5	
subitem not at all important	○	○	○	◉	○	essential

Clear selection

Does your paper address subitem 22-i? *

Copy and paste relevant sections from the manuscript (include quotes in quotation marks "like this" to indicate direct quotes from your manuscript), or elaborate on this item by providing additional information not in the ms, or briefly explain why the item is not applicable/relevant for your study

"This trial evaluated the effects of group-based VR-CBT with 360° video exposure versus traditional in-vivo group CBT for treating SAD and agoraphobia in a naturalistic clinical setting. Both groups showed reductions in symptoms, with no statistically significant differences observed in primary, secondary, or exploratory outcomes. "

22-ii) Highlight unanswered new questions, suggest future research

Highlight unanswered new questions, suggest future research.

	1	2	3	4	5	
subitem not at all important	○	○	◉	○	○	essential

Clear selection

Does your paper address subitem 22-ii?

Copy and paste relevant sections from the manuscript (include quotes in quotation marks "like this" to indicate direct quotes from your manuscript), or elaborate on this item by providing additional information not in the ms, or briefly explain why the item is not applicable/relevant for your study

"The feasibility challenges encountered underscore the need to carefully evaluate both the benefits and limitations of VR technology before clinical implementation. Future research could explore the VR technologies that account for the group therapy context, such as multi-user VR or augmented reality."

20) Trial limitations, addressing sources of potential bias, imprecision, and, if relevant, multiplicity of analyses

20-i) Typical limitations in ehealth trials

Typical limitations in ehealth trials: Participants in ehealth trials are rarely blinded. Ehealth trials often look at a multiplicity of outcomes, increasing risk for a Type I error. Discuss biases due to non-use of the intervention/usability issues, biases through informed consent procedures, unexpected events.

	1	2	3	4	5	
subitem not at all important	○	○	●	○	○	essential

Clear selection

Does your paper address subitem 20-i? *

Copy and paste relevant sections from the manuscript (include quotes in quotation marks "like this" to indicate direct quotes from your manuscript), or elaborate on this item by providing additional information not in the ms, or briefly explain why the item is not applicable/relevant for your study

"However, due to the limited statistical power of the study, these results must be interpreted with caution. We cannot conclude that VR-CBT and traditional CBT are equally effective, nor can we exclude the possibility of differences in efficacy between the two treatments. "

"Frequent technical malfunctions and limited time for in-session troubleshooting also impacted feasibility. These factors likely contributed to the low mean exposure time per VR-CBT group (85 minutes)."

21) Generalisability (external validity, applicability) of the trial findings

NPT: External validity of the trial findings according to the intervention, comparators, patients, and care providers or centers involved in the trial

21-i) Generalizability to other populations

Generalizability to other populations: In particular, discuss generalizability to a general Internet population, outside of a RCT setting, and general patient population, including applicability of the study results for other organizations

	1	2	3	4	5	
subitem not at all important	○	○	○	○	○	essential

Does your paper address subitem 21-i?

Copy and paste relevant sections from the manuscript (include quotes in quotation marks "like this" to indicate direct quotes from your manuscript), or elaborate on this item by providing additional information not in the ms, or briefly explain why the item is not applicable/relevant for your study

We took efforts to conduct the study in a naturalistic setting and population. We state this in the discussion and elsewhere in the paper.

21-ii) Discuss if there were elements in the RCT that would be different in a routine application setting

Discuss if there were elements in the RCT that would be different in a routine application setting (e.g., prompts/reminders, more human involvement, training sessions or other co-interventions) and what impact the omission of these elements could have on use, adoption, or outcomes if the intervention is applied outside of a RCT setting.

	1	2	3	4	5	
subitem not at all important	○	○	◉	○	○	essential

Clear selection

Does your paper address subitem 21-ii?

Copy and paste relevant sections from the manuscript (include quotes in quotation marks "like this" to indicate direct quotes from your manuscript), or elaborate on this item by providing additional information not in the ms, or briefly explain why the item is not applicable/relevant for your study

Our study was conducted in a routine application setting. We discuss feasibility issues encountered.

OTHER INFORMATION

23) Registration number and name of trial registry

Does your paper address CONSORT subitem 23? *

Copy and paste relevant sections from the manuscript (include quotes in quotation marks "like this" to indicate direct quotes from your manuscript), or elaborate on this item by providing additional information not in the ms, or briefly explain why the item is not applicable/relevant for your study

"ClinicalTrials.gov as NCT03845101"

24) Where the full trial protocol can be accessed, if available

Does your paper address CONSORT subitem 24? *

Cite a Multimedia Appendix, other reference, or copy and paste relevant sections from the manuscript (include quotes in quotation marks "like this" to indicate direct quotes from your manuscript), or elaborate on this item by providing additional information not in the ms, or briefly explain why the item is not applicable/relevant for your study

We refer to the trial protocol multiple times in the paper

25) Sources of funding and other support (such as supply of drugs), role of funders

Does your paper address CONSORT subitem 25? *

Copy and paste relevant sections from the manuscript (include quotes in quotation marks "like this" to indicate direct quotes from your manuscript), or elaborate on this item by providing additional information not in the ms, or briefly explain why the item is not applicable/relevant for your study

The Novo Nordisk Foundation awarded the SoREAL trial 5,000,000 DKK [NNF17OC0027780]. Additionally, the trial received a grant of 3,517,500 DKK from TrygFonden [ID: 146169]. The project is fully independent of both the Novo Nordisk Foundation and TrygFonden. Neither funding body influenced the study's design, data collection, analysis, interpretation, or manuscript preparation.

X27) Conflicts of Interest (not a CONSORT item)

X27-i) State the relation of the study team towards the system being evaluated

In addition to the usual declaration of interests (financial or otherwise), also state the relation of the study team towards the system being evaluated, i.e., state if the authors/evaluators are distinct from or identical with the developers/sponsors of the intervention.

	1	2	3	4	5	
subitem not at all important	○	○	○	●	○	essential

Clear selection

Does your paper address subitem X27-i?

Copy and paste relevant sections from the manuscript (include quotes in quotation marks "like this" to indicate direct quotes from your manuscript), or elaborate on this item by providing additional information not in the ms, or briefly explain why the item is not applicable/relevant for your study

"Conflict of Interest Statement
The authors declare no conflicts of interest related to this study."

About the CONSORT EHEALTH checklist

As a result of using this checklist, did you make changes in your manuscript? *

- ○ yes, major changes
- ● yes, minor changes
- ○ no

What were the most important changes you made as a result of using this checklist?

Improved abstract and outcome section

How much time did you spend on going through the checklist INCLUDING making changes in your manuscript *

I spent aprox. five hours

As a result of using this checklist, do you think your manuscript has improved? *

- (selected) yes
- no
- Other:

Would you like to become involved in the CONSORT EHEALTH group?

This would involve for example becoming involved in participating in a workshop and writing an "Explanation and Elaboration" document

- yes
- (selected) no
- Other:

Clear selection

Any other comments or questions on CONSORT EHEALTH

Your answer

STOP - Save this form as PDF before you click submit

To generate a record that you filled in this form, we recommend to generate a PDF of this page (on a Mac, simply select "print" and then select "print as PDF") before you submit it.

When you submit your (revised) paper to JMIR, please upload the PDF as supplementary file.

Don't worry if some text in the textboxes is cut off, as we still have the complete information in our database. Thank you!

Final step: Click submit !

Click submit so we have your answers in our database!

Submit Clear form

Never submit passwords through Google Forms.

This content is neither created nor endorsed by Google. - Terms of Service - Privacy Policy

Does this form look suspicious? Report

Google Forms
